# Incidence of immune effector cell-associated neurotoxicity among patients treated with CAR T-cell therapy for hematologic malignancies: systematic review and meta-analysis

**DOI:** 10.3389/fneur.2024.1392831

**Published:** 2024-10-15

**Authors:** Min Woo Han, So Yeong Jeong, Chong Hyun Suh, Hyesun Park, Jeffrey P. Guenette, Raymond Y. Huang, Kyung Won Kim, Dok Hyun Yoon

**Affiliations:** ^1^University of Ulsan College of Medicine, Seoul, Republic of Korea; ^2^Department of Radiology, Seoul National University Bundang Hospital, Seoul National University College of Medicine, Seongnam, Republic of Korea; ^3^Department of Radiology and Research Institute of Radiology, Asan Medical Center, University of Ulsan College of Medicine, Seoul, Republic of Korea; ^4^Department of Imaging, Dana-Farber Cancer Institute, Harvard Medical School, Boston, MA, United States; ^5^Division of Neuroradiology, Brigham and Women's Hospital, Dana-Farber Cancer Institute, Harvard Medical School, Boston, MA, United States; ^6^Department of Oncology, Asan Medical Center, University of Ulsan College of Medicine, Seoul, Republic of Korea

**Keywords:** CAR T-cell, immunotherapy, immune effector cell-associated neurotoxicity syndrome, neurotoxicity, hematologic malignancies

## Abstract

**Objectives:**

We aim to assess the pooled incidence of immune effector cell-associated neurotoxicity syndrome (ICANS) in clinical trials and real-world studies of chimeric antigen receptor (CAR) T-cell therapy for hematologic malignancy and compare the incidences among different agents.

**Methods:**

The PubMed, Embase, and Web of Science databases were searched for clinical trials and real-world studies. An inverse-variance weighting model was used to calculate pooled incidences and subgroup analyses. Multivariable analysis was conducted using binomial-normal modeling.

**Results:**

Seventy-five trials comprising 3,184 patients were included. The overall pooled incidence was 26.9% (95% CI, 21.7–32.7%) for all-grade and 10.5% (95% CI, 8.1–13.6%) for high-grade ICANS. In subgroup analysis, cohorts with anti-CD19 drugs had significantly higher ICANS incidences than cohorts with other agents. The multivariable analysis demonstrated higher odds of ICANS in anti-CD19 drug studies for high-grade (OR, 4.6) compared to anti-BCMA drug studies. In 12 real-world studies, studies used axicabtagene ciloleucel with CD28 (54.0% all-grade, 26.4% high-grade) exhibited significantly higher rates of all-grade and high-grade ICANS than studies using tisagenlecleucel with 4-1BB (17.2% all-grade, 6.1% high-grade).

**Conclusions:**

The overall incidences of ICANS with CAR T-cell therapy were 26.9% for all-grade and 10.5% for high-grade. Compared with other agents, patients with anti-CD19 drugs had a significantly increased risk of developing high-grade ICANS. Therefore, careful monitoring of ICANS should be considered for patients undergoing CAR T-cell therapy.

## Highlights

- The pooled incidence of ICANS with CAR T-cell therapy was 26.9% (95% CI, 21.7–32.7%) for all-grade and 10.5% (95% CI, 8.1–13.6%) for high-grade.
- Univariable meta-regression analysis showed that leukemia patients (OR, 4.7; 95% CI, 1.5–14.2; *P* = 0.007) and lymphoma patients (OR, 3.1; 95% CI, 1.1–9.1; *P* = 0.036) had higher odds of all-grade ICANS compared with patients with multiple myeloma.
- Multivariable meta-regression analysis showed that patients treated with anti-CD19 drugs had higher odds for all-grade (OR, 2.7; 95% CI, 1.0–7.7; *P* = 0.057) and high-grade (OR, 4.6; 95% CI, 1.5–13.7; *P* = 0.008) ICANS compared with patients treated with anti-BCMA drugs.

## Introduction

Chimeric antigen receptor (CAR) T-cell therapy is promising immunotherapy for hematologic malignancies (1–3). Studies have shown a high response rate to CAR T-cell therapy with remission rates of up to 80% or more in patients with relapsed or refractory hematologic malignancies (4–6). To date, six CAR T-cell therapies targeting the CD19 antigen or B-cell maturation antigen (BCMA) have received approval from the United States Food and Drug Administration (FDA): tisagenlecleucel, axicabtagene ciloleucel, brexucabtagene autoleucel, lisocabtagene maraleucel, idecabtagene vicleucel, and ciltacabtagene autoleucel (7–9). Toxicities associated with CAR T-cell therapy, including cytokine-release syndrome (CRS) and neurotoxicity, have been reported as adverse events in almost all clinical trials (10–13).

Neurotoxicity, what is termed “immune effector cell-associated neurotoxicity syndrome (ICANS),” is the second most common adverse event following CRS. The incidence of ICANS has been reported to range widely, from 5 to 42%, in clinical trials (1, 14–20), but the exact incidence of ICANS among patients undergoing CAR T-cell therapy has not been systematically investigated in large-scale datasets. Furthermore, although it has been assumed that incidences of ICANS differ according to the type of agent used or other factors such as the co-stimulatory domain or number of agents, there is no concrete evidence to support this hypothesis or demonstrate the magnitude of the differences.

We, therefore, performed a systematic review and meta-analysis of the incidence of ICANS in clinical trials and real-world studies of CAR T-cell therapies for hematologic malignancies. Our aim is to determine if there are differences in the incidence of ICANS among cohorts with various underlying diseases, treated with agents targeting different antigens, and utilizing different co-stimulatory domains. The null hypothesis was that there would be no differences in the incidence of ICANS across these cohorts.

Clinical trials of CAR T-cell therapies for hematologic malignancies, until May 28, 2022 were included in the study. The pooled incidence of ICANS was calculated using the inverse-variance weighting method.

## Materials and methods

We followed the Preferred Reporting Items for Systematic Reviews and Meta-Analysis (PRISMA) guidelines (21, 22); the completed checklist is provided in Supplementary Table 1.

### Search methods and study selection

The PubMed, Embase, and Web of Science databases were searched for published clinical trials of CAR T-cell therapies administered to patients with hematologic malignancies until May 28, 2022. The search terms were formulated using “car t cell therapy” and “neurotoxicity” as keywords. Details and specific search queries are described in the Supplementary Tables 2–4. This study was registered in the international prospective register of systematic reviews (PROSPERO; CRD4202233390960).

The inclusion criteria were as follows: (1) clinical trials with patients treated with CAR T-cell therapies for hematologic malignancies and (2) detailed data sufficient to assess the incidence of ICANS. The exclusion criteria were as follows: (1) conference abstracts, review articles, letters, editorials, comments, notes, short surveys, or chapters; (2) studies other than clinical trials; (3) studies not reporting on CAR T-cell therapy; (4) studies not discussing ICANS; (5) studies with patient numbers below 10; (6) study protocols; (7) studies with patient cohorts overlapping with those of other studies; and (8) summaries of other studies.

For additional analysis of the incidence of ICANS in real-world studies, an additional computerized search of the literature was performed using the PubMed and Embase databases for published real-world studies of CAR T-cell therapies until August 13, 2022. The search terms and inclusion/exclusion criteria were identical to the analysis for previous clinical trials. The added inclusion criterion was (1) studies in the real-world clinical setting, and the added exclusion criterion was (1) studies with patient numbers below 100.

**Figure 1:**
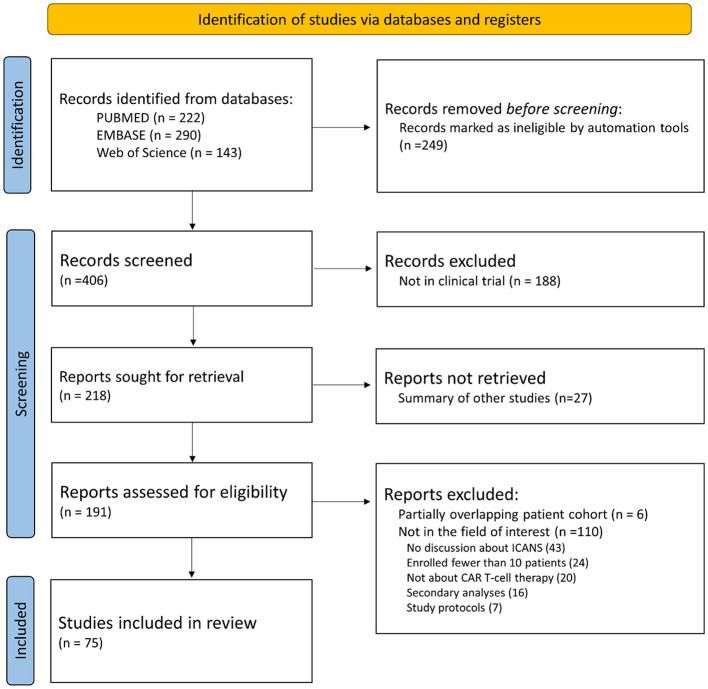
Flow diagram of clinical trial study inclusion.

The 75 eligible studies of clinical trials comprised of 3,184 patients were included in our analysis (Figure 1), and 12 eligible studies of real-world studies comprised of 3,403 patients were included for additional analysis for real-world clinical settings.

### Data extraction

From the each eligible article, we extracted data indicating the numbers of patients who developed ICANS of all grade and high-grade (grade 3 or higher), and the numbers of ICANS-related deaths (grade 5). Different articles referred to ICANS in varying ways; we included reports on “neurotoxicity,” “neurologic events,” or “neurologic symptoms.” In addition, the phase of each clinical trial, NCT numbers, types of specific CAR T-cells used, and the included patients' diseases were recorded. Specifically, the CAR T-cell types included all landmark treatments with FDA approval (axicabtagene ciloleucel, brexucabtagene autoleucel, lisocabtagene maraleucel, tisagenlecleucel, idecabtagene vicleucel, and ciltacabtagene autoleucel); we also included various types of CAR T-cells currently waiting for FDA approval. We focused on the targets of CAR T-cells rather than the brand of each drug. Two reviewers (M.W.H and S.Y.J) performed the data extraction, with an independent review by an additional reviewer (C.H.S); when discrepancies were identified between the data extracted by the two reviewers, they first engaged in a discussion to understand the source of the disagreement and to try to resolve it collaboratively. If the discussion did not lead to a consensus, they referred to the predefined data extraction protocol to ensure alignment with the study's objectives and criteria. Throughout this process, all discrepancies and their resolutions were thoroughly documented to maintain transparency and ensure the integrity of the review.

### Quality assessment

We assessed the risk of bias for each study using the Cochrane risk of bias tool for randomized controlled trials (23). The tool evaluates seven domains including random sequence generation, allocation concealment, blinding of participants and personnel, blinding of outcome assessment, incomplete outcome data, selective reporting, and others to assess selection, performance, detection, attrition, and reporting biases. Based on available information, two independent reviewers (M.W.H and S.Y.J) scored each domain as high or low risk or unclear. Disagreements were resolved by discussion.

In addition, the quality of evidence from the pooled results were evaluated using the Grading of Recommendations Assessment, Development, and Evaluation (GRADE) system (24).

### Statistical methods

The pooled incidence of all-grade and high-grade ICANS was obtained using an inverse-variance weighting model (25). Heterogeneity was evaluated with Cochran's Q test and Higgins inconsistency index (*I*^2^) test, with values >50% indicating substantial heterogeneity. Publication bias was evaluated and recorded using Egger's test and funnel plots (26).

The pooled incidence of ICANS was also obtained for each subgroup classified according to CAR T-cell agent, patient disease, trial phase, number of used drugs (monotherapy vs. combination therapy), co-stimulatory domain, lymphodepletion strategy, and involvement or non-involvement of the CNS. Univariable meta-regression analyses were conducted to assess the associations between each of the study-level covariates and the incidence of ICANS. Multivariable analysis was performed using binomial-normal modeling (27, 28). To test if study-level covariates as moderators had statistical effects in the meta-regression, the regression coefficient was obtained to estimate the intervention effect on each subgroup from a reference group. All statistical analyses were performed using the “meta” package of R version 4.0.4 (R Foundation for Statistical Computing, Vienna, Austria) (29).

## Results

### Literature search

Initially, 222 articles from PubMed, 290 articles from Embase, and 143 articles from Web of Science were screened using keywords. Of the 655 articles, 249 were excluded by an automation tool and by a review of the article titles to eliminate duplication, conference abstracts, reviews, letters, editorials, comments, notes, short surveys, and chapters. The complete texts of the remaining 406 articles were retrieved, and 331 studies were further excluded after reviewing the full texts (188 articles were not clinical trials, 27 were summaries of other studies, six had partially overlapping patient cohorts, 43 did not discuss ICANS, 24 enrolled fewer than 10 patients, 20 were not about CAR T-cell therapy, 16 presented secondary analyses, and seven were about study protocols). The remaining 75 eligible studies comprised of 3,184 patients were included (1, 4, 5, 14–20, 30–94).

For an additional search for analysis of the incidence of ICANS in real-world clinical settings, 12 studies comprised of 3,403 patients were included in the analysis for real-world studies (95–106). The detailed literature search for analysis of real-world studies is provided in Supplementary material.

### Risk of bias

A risk of bias assessment was performed for each study (Supplementary Figure 1). Based on available information, each criterion was scored by two independent reviewers as high risk, low risk, or unclear. Disagreements were resolved by discussion. Details of trial evaluations are detailed in Supplementary Table 5.

### Characteristics of the included studies

Table 1 summarizes the characteristics of the included studies. Among the 75 eligible trial cohorts, 42 cohorts (1, 14, 16, 18–20, 30, 31, 33–36, 39, 40, 45, 47, 49, 50, 52, 53, 55, 56, 58, 60–64, 66, 69–81) (56.0%) were phase 1 trials, 21 (4, 5, 15, 17, 42, 43, 51, 54, 57, 59, 65, 67, 68, 82–86, 88, 89, 91) (28.0%) were phase 2 trials, and one (92) (1.3%) was a phase 3 trial. Nine studies (32, 37, 38, 41, 44, 46, 48, 87, 93) employed combined designs for phase 1/2 trials; these studies (combined phase 1/2 or 1b/2 trials) were included in the analysis as phase 2 trials. The remaining two studies (90, 94) (2.7%) did not mention the trial phase. Different studies used different types of CAR T-cells, and we classified each drug according to its specific target. The FDA-approved agents all target CD19, except idecabtagene vicleucel and ciltacabtagene autoleucel, which target BCMA. Among the 75 cohorts, 47 (62.7%) (1, 4, 5, 14, 16, 17, 31, 33, 35–38, 40, 41, 43, 47–50, 53–55, 57–63, 66, 68, 69, 72, 74, 75, 77, 78, 82–86, 88, 91–94) used agents that target CD19, nine (12.0%) (20, 32, 34, 39, 42, 45, 52, 56, 71) used agents that target BCMA, two (2.7%) (18, 44) used agents that target CD22, and 17 others (22.7%) (15, 19, 30, 46, 51, 64, 65, 67, 70, 73, 76, 79–81, 87, 89, 90) used agents that target various proteins, such as CD7, CD20, CD28, CD30, or NKG2D. Moreover, 61 of the 75 cohorts (81.3%) used single agents (anti-CD19, anti-BCMA, anti-CD22, anti-CD30, anti-CD7, or anti-NKG2D), 14 (18.7%) used combinations of agents (anti-CD19+anti-BCMA, anti-CD19+anti-20, anti-CD19+anti-22, anti-CD19+anti-28, or anti-BCMA+anti-38). Additional details about agents, doses and CD4:CD8 ratios for each cohort are listed in Supplementary Table 6.

**Table 1:** Characteristics of all eligible trial cohorts.

**Study characteristics**		**Cohorts (*****N*** = **75)**	
Phase	I	42	(56.0%)^*^
	II	30	(40.0%)
	III	1	(1.3%)
	Unknown	2	(2.7%)
CAR T-cell targets	CD19	47	(62.7%)
	CD22	2	(2.7%)
	BCMA	9	(12.0%)
	Mixed/other	17	(22.7%)
Patient disease	Leukemia	23	(30.7%)
	Lymphoma	32	(42.7%)
	Multiple myeloma	12	(16.0%)
	Mixed/other	8	(10.7%)
Number of agents	Single-agent	61	(81.3%)
	Combination of agent	14	(18.7%)
Co-stimulatory domain	4-1BB	43	(57.3%)
	CD28	22	(29.3%)
	Combination	4	(5.3%)
	Mixed/other	6	(8.0%)
Lymphodepletion strategy	Fludarabine + cyclophosphamide	58	(77.3%)
	Fludarabine only	1	(1.3%)
	Cyclophosphamide only	3	(4.0%)
	BEAM protocol	2	(2.7%)
	Others	11	(14.7%)
CNS involvement	Included	25	(33.3%)
	Included with no actual involvement	4	(5.3%)
	Excluded	25	(33.3%)
	No information	21	(28.0%)

^*^Nine phase I/II or phase Ib/II studies were included as phase II trials.

BCMA, B-cell maturation antigen; BEAM, carmustine, etoposide, cytarabine, and melphalan; CAR, chimeric antigen receptor; CNS, central nervous system.

Among the 75 trial cohorts, 24 (32.0%) included treatment for leukemia, 32 (42.7%) for lymphoma, and 12 (16.0%) for multiple myeloma. In seven articles (9.3%), patients with various diseases were pooled or the diseases treated were not stated.

Four kinds of grading schemes of ICANS were used across studies: the National Cancer Institute Common Terminology Criteria for Adverse Events, the American Society for Transplantation and Cellular Therapy scale, the American Society for Blood and Marrow Transplantation consensus, and the MD Anderson Cancer Center Scale CAR-T-cell-related encephalopathy syndrome grading system. Additional details about the ICANS grading schemes for each cohort are listed in Supplementary Table 6.

Among the 12 included studies for additional analysis of the real-world studies, seven studies included patients used agents of axicabtagene ciloleucel and tisagenlecleucel, respectively (95, 96, 98, 99, 102, 105, 106). Three studies included patients who used axicabtagene ciloleucel (100, 103, 104), and two had those who used tisagenlecleucel (97, 101). All patients treated with axicabtagene ciloleucel used CD28 as a co-stimulatory domain, and all patients treated with tisagenlecleucel used 4-1BB as a co-stimulatory domain.

### Pooled incidence of neurotoxicity among patients undergoing CAR T-cell therapy

**Figure 2:**
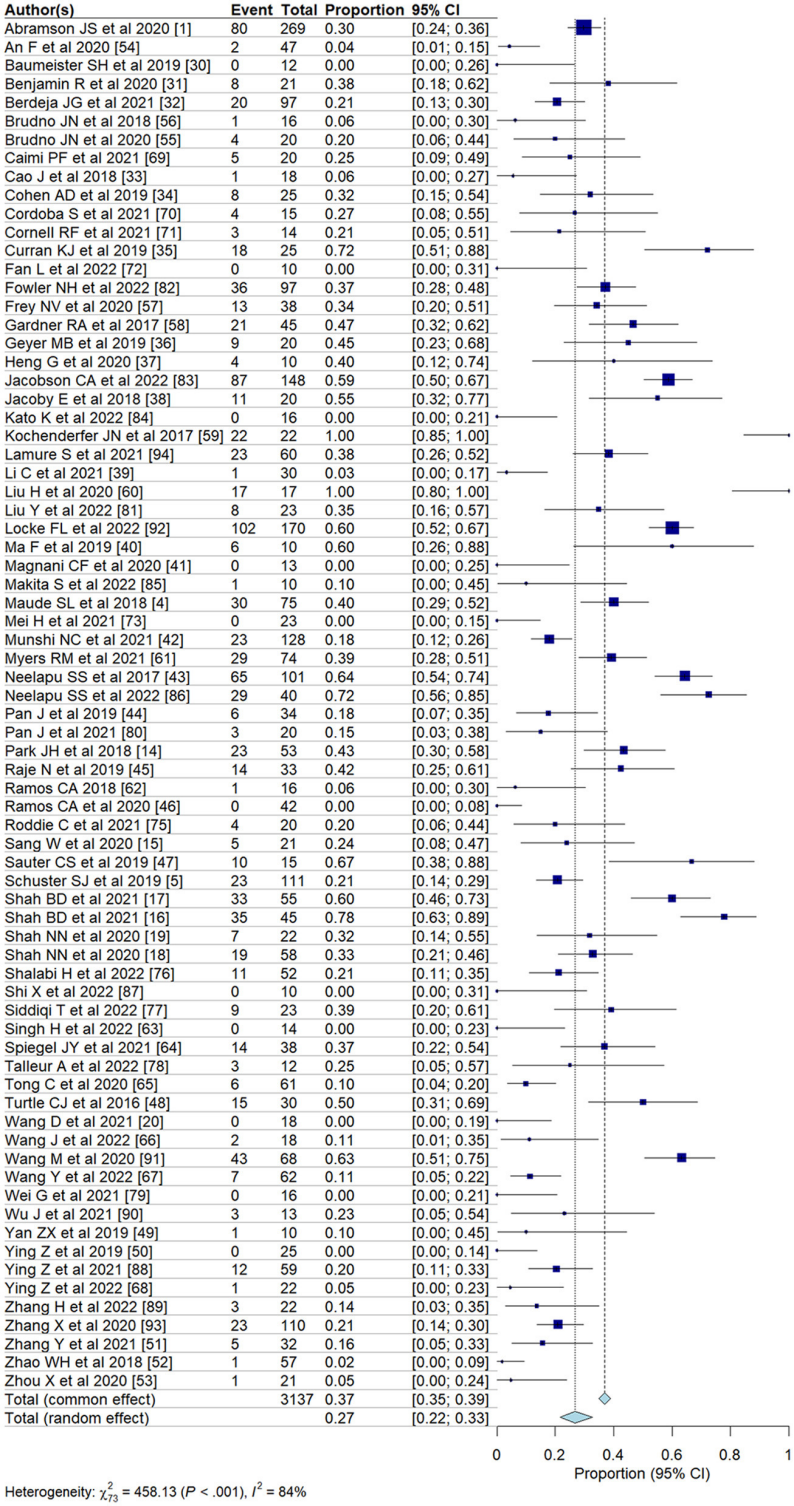
Forest plots showing the pooled incidence of all-grade ICANS in clinical trials.

**Figure 3:**
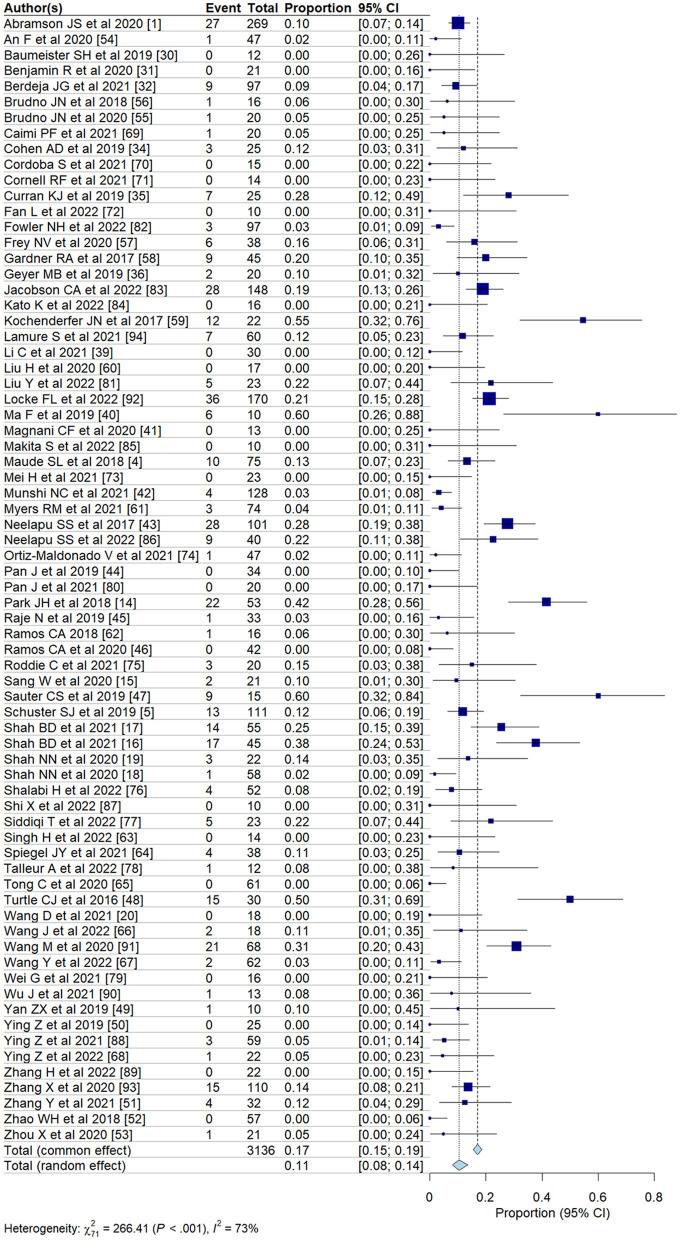
Forest plots showing the pooled incidence of high-grade ICANS in clinical trials.

We evaluated the incidence of all-grade and high-grade ICANS after CAR T-cell infusion in a total of 75 cohorts of patients. The overall pooled incidence, evaluated with a random effects model, was 26.9% (95% CI, 21.7–32.7%; Figure 2) for all grades and 10.5% (95% CI, 8.1–13.6%; Figure 3) for high-grade ICANS (Table 2). Heterogeneity was observed in both all-grade and high-grade ICANS (*I*^2^ = 84.1% and *I*^2^ = 73.3%, respectively). Publication bias likely occurred in both all-grade and high-grade ICANS analyses (*P* < 0.01; Supplementary Figures 2, 3). Three cases of grade 5 ICANS were reported across cohorts, one in a study using a brexucabtagene autoleucel agent in leukemia patients, one in a cohort of multiple myeloma patients using a ciltacabtagene autoleucel agent, and one in leukemia patients using an anti-CD19 agent.

**Table 2:** Results of the multiple subgroup analysis on the incidence of ICANS after CAR T-cell therapy.

		**All-grade ICANS**		**High-grade ICANS (**≥**grade 3)**	
		**Studies (** ***N*** **)**	**Incidence (%) (95% CI)**	**Studies (** ***N*** **)**	**Incidence (%) (95% CI)**
Overall		75	26.9 (21.7–32.7)^*^	72^†^	10.5 (8.1–13.6)^*^
**Subgroup**					
CAR T-cell targets	BCMA	9	15.1 (7.7–27.7)^*^	9	5.2 (2.9–9.3)
	CD22	2	25.8 (13.6–43.5)^*^	2	1.6 (0.3–7.6)
	CD19	46	34.8 (27.2–43.2)^*^	44	14.7 (10.9–19.5)^*^
Disease	Multiple myeloma	12	15.1 (9.1–24.0)^*^	12	4.9 (3.0–8.1)
	Lymphoma	32	27.5 (18.9–38.2)^*^	32	11.3 (7.8–16.1)^*^
	Leukemia	23	36.5 (27.9–46.1)^*^	20	15.5 (9.6–24.0)^*^
Phase	1	40	24.6 (18.3–32.2)^*^	40	10.0 (6.8–14.4)^*^
	2	29	25.9 (18.4–35.0)^*^	27	10.1 (6.7–14.8)^*^
Number of agents	Single-agent	60	28.9 (22.6–36.1)^*^	58	11.2 (8.3–15.0)^*^
	Combination of agents	14	19.7 (14.4–26.4)	14	8.6 (5.7–12.8)
Co-stimulatory domain	4-1BB	42	26.4 (21.0–32.5)^*^	42	10.1 (7.1–14.0)^*^
	CD28	22	29.6 (19.6–42.1)^*^	20	10.4 (6.2–17.0)^*^
	Combination	4	14.8 (2.5–54.2)^*^	4	10.0 (2.4–33.6)
Lymphodepletion	Fludarabine + cyclophosphamide	57	25.5 (20.0–31.8)^*^	57	10.0 (7.3– 13.6)^*^
	BEAM	2	64.0 (46.2–78.7)	1	28.0 (14.0–48.2)
	Cyclophosphamide	3	54.4 (28.0–78.6)^*^	2	23.5 (7.4–54.2)^*^
	Fludarabine	1	58.8 (50.7–66.4)	1	18.9 (13.4–26.0)
CNS involvement	Included	25	30.9 (22.4–41.0)^*^	23	13.2 (9.0–19.0)^*^
	Included but no involvement	4	29.9 (8.2–67.0)^*^	4	15.5 (6.8–31.7)^*^
	Excluded	25	25.8 (17.3–36.6)^*^	25	10.6 (6.6–16.6)^*^
	No information	20	22.0 (13.7–33.5)^*^	20	6.5 (3.5–11.8)^*^

CI, confidence interval; ICANS, immune effector cell-associated neurotoxicity syndrome.

^*^I^2^ > 50% indicating substantial heterogeneity.

^†^Three studies not reporting on high-grade ICANS were excluded. BEAM: carmustine for day −6, etoposide from days −5 to −2, cytarabine from days −5 to −2, and melphalan for day −1.

To address the high heterogeneity observed in the incidence of all-grade and high-grade ICANS, we conducted a sensitivity analysis by removing ~15% of the outlier studies (11 out of 75 studies). This adjustment resulted in a substantial reduction in heterogeneity, with the *I*^2^ for all-grade ICANS decreasing from 84 to 67%, and for high-grade ICANS from 73 to 43%, while maintaining the overall trend of our results (Supplementary Figure 4).

### Subgroup analyses

We evaluated the incidence of ICANS classified according to target agent (Table 2). The pooled incidences associated with anti-BCMA agents were 15.1% (95% CI, 7.7–27.7%) for all-grade and 5.2% (95% CI, 2.9–9.3%) for high-grade ICANS. The pooled incidences associated with anti-CD22 agents were 25.8% (95% CI, 13.6–43.5%) for all-grade and 1.6% (95% CI, 0.3–7.6%) for high-grade ICANS. The pooled incidences associated with anti-CD19 agents were 34.8% (95% CI, 27.2–43.2%) for all-grade and 14.7% (95% CI, 10.9–19.5%) for high-grade ICANS. Agents targeting CD19 showed significantly higher rates of both all-grade and high-grade ICANS than agents targeting CD22 or BCMA (*P* < 0.05 for all-grade and high-grade).

In subgroups classified by patient disease, the pooled incidences for patients with multiple myeloma were 15.1% (95% CI, 9.1–24.0%) for all-grade and 4.9% (95% CI, 3.0–8.1%) for high-grade ICANS. For patients with lymphoma, the pooled incidences were 27.5% (95% CI, 18.9–38.2%) for all-grade and 11.3% (95% CI, 7.8–16.1%) for high-grade ICANS. Finally, for patients with leukemia, the pooled incidences were 36.5% (95% CI, 27.9–46.1%) for all-grade and 15.5% (95% CI, 9.6–24.0%) for high-grade ICANS. Patients with leukemia had significantly higher rates of both all-grade and high-grade ICANS than patients with lymphoma or multiple myeloma (*P* < 0.05 for all-grade and high-grade).

In subgroups classified according to the number of agents, cohorts using a single agent exhibited higher rates of all-grade ICANS (*P* = 0.05) than cohorts using combinations of agents. In subgroups classified by co-stimulatory domain, the pooled incidences for 4-1BB, evaluated with a random effects model, were 26.4% (95% CI, 21.0–32.5%) for all-grade and 10.1% (95% CI, 7.1–14.0%) for high-grade ICANS. For CD28, the pooled incidences were 29.6% (95% CI, 19.6–42.1%) for all-grade and 10.4% (95% CI, 6.2–17.0%) for high-grade ICANS.

### Univariable and multivariable meta-regression analyses

The univariable meta-regression analyses yielded significantly higher odds for all-grade [odds ratio [OR], 3.7; 95% CI, 1.2–11.9; *P* = 0.029] and high-grade (OR, 4.9; 95% CI, 1.6–14.7; *P* = 0.006) ICANS for the cohorts treated with anti-CD19 drugs than for those treated with anti-BCMA drugs (Tables 3, 4). The cohorts consisting of leukemia patients had significantly higher odds for all-grade (OR, 4.7; 95% CI, 1.5–14.2; *P* = 0.007) and high-grade (OR, 5.9; 95% CI, 1.8–19.0; *P* = 0.003) ICANS than those consisting of multiple myeloma patients. The cohorts of lymphoma patients also had significantly higher odds for all-grade (OR, 3.1; 95% CI, 1.1–9.1; *P* = 0.036) and high-grade (OR, 3.9; 95% CI, 1.3–11.8; *P* = 0.017) ICANS than the cohorts of multiple myeloma patients. No significant differences in ICANS incidences were observed between cohorts with different trial phases, therapy types, co-stimulatory domains, and CNS involvement or non-involvement.

**Table 3:** Results of the univariable and multivariable meta-regression on the incidence of all-grade ICANS.

		**All-grade ICANS**			
		**Univariable meta-regression**		**Multivariable meta-regression**	
**Variable**		**Odds ratio (95% CI)**	***P*** **-value**	**Odds ratio (95% CI)**	***P*** **-value**
Drug agents	Anti-BCMA	REF		REF	
	Anti-CD22	2.6 (0.2–27.7)	0.428	2.3 (0.3–17.8)	0.423
	Anti-CD19	3.7 (1.2–11.9)	0.029	2.7 (1.0–7.7)	0.057
Disease	Multiple myeloma	REF			
	Lymphoma	3.1 (1.1–9.1)	0.036		
	Leukemia	4.7 (1.5–14.2)	0.007		
Phase	1	REF		REF	
	2	1.2 (0.6–2.5)	0.603	1.3 (0.6–2.9)	0.448
Number of agents	Single agent	REF			
	Combination of agents	0.5 (0.2–1.3)	0.138		
Co-stimulatory domain	4-1BB	REF			
	CD28	1.2 (0.5–2.9)	0.609		
	Combination	0.5 (0.1–3.0)	0.426		
CNS involvement	Included	REF			
	Included without actual involvement	0.9 (0.2–5.4)	0.927		
	Excluded	0.8 (0.3–2.0)	0.620		
	No information	0.7 (0.3–1.8)	0.450		

REF, the reference group.

**Table 4:** Results of the univariable and multivariable meta-regression on the incidence of high-grade ICANS.

		**High-grade neurotoxicity**			
		**Univariable meta-regression**		**Multivariable meta-regression**	
**Variable**		**Odds ratio (95% CI)**	***P*** **-value**	**Odds ratio (95% CI)**	***P*** **-value**
Drug agents	Anti-BCMA	REF		REF	
	Anti-CD22	0.3 (0.01–4.8)	0.356	0.3 (0.01–4.7)	0.351
	Anti-CD19	4.9 (1.6–14.7)	0.006	4.6 (1.5–13.7)	0.008
Disease	Multiple myeloma	REF			
	Lymphoma	3.9 (1.3–11.8)	0.017		
	Leukemia	5.9 (1.8–19.0)	0.003		
Phase	1	REF		REF	
	2	1.1 (0.5–2.4)	0.831	1.2 (0.6–2.5)	0.687
Number of agents	Single agent	REF			
	Combination of agents	0.5 (0.2–1.3)	0.158		
Co-stimulatory domain	4-1BB	REF			
	CD28	1.0 (0.4–2.4)	0.992		
	Combination	1.0 (0.2–5.9)	0.966		
CNS involvement	Included	REF			
	Included without actual involvement	1.0 (0.2–5.1)	0.997		
	Excluded	0.7 (0.3–1.6)	0.366		
	No information	0.4 (0.1–1.0)	0.048		

The multivariable meta-regression analyses showed high levels of coexistence and concurrence for types of drug agents and diseases. Diseases were excluded from the multivariable analysis to avoid multi-collinearity. The cohorts treated with anti-CD19 drugs had higher odds for all-grade (OR, 2.7; 95% CI, 1.0–7.7; *P* = 0.057) ICANS than cohorts treated with anti-BCMA drugs, with borderline significance. The cohorts treated with anti-CD19 drugs had significantly higher odds for high-grade (OR, 4.6; 95% CI, 1.5–13.7; *P* = 0.008) ICANS than cohorts treated with anti-BCMA drugs.

### Evidence of the critical outcomes

The GRADE system was used to assess the certainty of evidence for the pooled incidence outcome (Table 5). Due to a high risk of bias in the included studies and a strongly suspected publication bias, the overall quality of evidence regarding the incidence of ICANS in patients receiving CAR-T cell therapy for hematologic malignancies was rated as moderate.

**Table 5:** Certainty of evidence for the incidence of all-grade and high-grade ICANS.

**No. of studies**	**Certainty assessment**						**Effect**			**Certainty**	**Importance**
	**Study design**	**Risk of bias**	**Inconsistency**	**Indirectness**	**Imprecision**	**Other considerations**	**No. of events**	**No. of individuals**	**Rate (95% CI)**		
**All-grade ICANS**											
75	Randomized trials	Serious	Serious^*^	Not serious	Not serious	Publication bias strongly suspected^†^ Strong association All plausible residual confounding would suggest spurious effect, while no effect was observed	1,034	3,184	Event rate 26.9 per 100 (21.7–32.7)	⊕⊕⊕○ Moderate^*†^	Critical
**High-grade ICANS**											
72	Randomized trials	Serious	Serious^*^	Not serious	Not serious	Publication bias strongly suspected^†^ Strong association All plausible residual confounding would suggest spurious effect, while no effect was observed	385	3,136	Event rate 10.5 per 100 (8.1–13.6)	⊕⊕⊕○ Moderate^*†^	Critical

GRADE Working Group certainty of evidence. High: very condifent that the true effect lies close to that of the estimate of the effect. Moderate: moderately confident in the effect estimate; the true effect is likely to be close to the estimate of the effect, but there is a possibility that it is substantially different. Low: our confidence in the effect estimate is limited; the true effect may be substantially different from the estimate of the effect. Very low quality: we have very little confidence in the effect estimate; the true effect is likely to be substantially different from the estimate of effect.

^*^Heterogeneity was observed in both all-grade and high-grade ICANS (I^2^ = 84.1 and 73.3%, respectively).

^†^Publication bias likely occurred in both all-grade and high-grade ICANS analyses (P <0.01; Supplementary Figures 2, 3).

GRADE, grading of recommendations assessment, development, and evaluation.

### Analysis of real-world studies

We evaluated the incidence of ICANS in real-world clinical data of 12 studies classified according to target agent (axicabtagene ciloleucel and tisagenlecleucel) (95–106). The pooled incidences for axicabtagene ciloleucel with CD28 were 54.0% (95% CI, 46.5–61.4%) for all-grade and 26.4% (95% CI, 21.3–32.2%) for high-grade ICANS. The pooled incidences for tisagenlecleucel with 4-1BB were 17.2% (95% CI, 13.7–21.4%) for all-grade and 6.1% (95% CI, 4.5–8.1%) for high-grade ICANS. Studies using axicabtagene ciloleucel with CD28 exhibited higher rates of all-grade and high-grade ICANS than studies using tisagenlecleucel with 4-1BB (*P* < 0.001 for all-grade and high-grade ICANS; Supplementary Figure 5). There were six deaths from ICANS (grade 5), and all cases were treated with axicabtagene ciloleucel with CD28 (95, 100, 102).

## Discussion

In the present meta-analyses, the overall ICANS incidence among patients undergoing CAR T-cell therapy for hematologic malignancies was 26.9% for all-grade and 10.5% for high-grade ICANS. In the subgroup analysis according to the type of agent, the cohorts with anti-CD19 drugs had significantly higher ICANS incidence than those with anti-BCMA drugs. The multivariable analysis also demonstrated significant higher odds of ICANS in anti-CD19 drug studies for high-grade (OR, 4.6; *P* = 0.008) ICANS compared with studies on anti-BCMA drugs. In real-world studies, studies using axicabtagene ciloleucel with CD28 (54.0% for all-grades, 26.4% for high-grades) exhibited significantly higher rates of ICANS than studies using tisagenlecleucel with 4-1BB (17.2% for all-grades, 6.1% for high-grades). The current study demonstrates that ICANS is not uncommon among patients undergoing CAR T-cell therapy, and our results provide further insights into risk factors for ICANS.

In terms of risk factors associated with ICANS in CAR T-cell therapies, previous studies reported that ICANS is significantly correlated with a high pretreatment disease burden and a higher peak CAR T-cell expansion (107, 108). However, it is difficult to predict which patients will develop ICANS, the timing of their symptoms, or their disease severity. Rubin et al. (103) developed a predictive scoring system for ICANS, which includes age, histologic subtype, maximum temperature, maximum C-reactive protein level, ferritin level, minimum white blood cell count, CRS severity, and CRS onset day. Our subgroup analysis by patient disease, drug agent, and the number of agents showed that patients with leukemia, and anti-CD19 drugs had significantly higher rates of ICANS than patients with other diseases, and drugs that have other targets. However, the high ICANS rate among leukemia patients may be attributable to confounding factors, given that CD19-targeting CAR T-cell drugs are more generally used to treat leukemia. The risk of ICANS in patients with preexisting neurologic comorbidities was controversial in previous studies (76, 109). However, in our study, the risk did not appear to be higher in groups with preexisting neurologic comorbidities.

On February 2022, the United States FDA approved ciltacabtagene autoleucel for treating adults with relapsed or refractory multiple myeloma, making it the sixth FDA-approved CAR T-cell agent. The ciltacabtagene autoleucel trials had reported atypical neurotoxicity (non-ICANS-related neurotoxicity) after the resolution of ICANS. Symptoms associated with non-ICANS neurotoxicity included parkinsonian symptoms, peripheral motor neuropathy, and cranial nerve palsies. Van Oekelen et al. (110) reported cases of multiple myeloma patients, enrolled in the CARTITUDE-1 trial, who developed a progressive movement disorder with features of parkinsonism 3 months after ciltacabtagene autoleucel infusion. These findings indicate that further research into non-ICANS-related neurotoxicity is needed.

Our multivariable analysis also demonstrated significantly increased odds of ICANS in the cohorts treated with anti-CD19 drugs for high-grade (OR, 4.6; *P* = 0.008) ICANS compared with cohorts treated with anti-BCMA drugs. These results indicate that anti-CD19 drugs are an independent risk factor for high-grade ICANS in patients undergoing CAR T-cell therapy. Multiple studies have explored the biological mechanisms of neurotoxicity in patients treated with CAR T-cells. The primary mechanism currently suggested is endothelial dysfunction and increased blood-brain barrier (BBB) permeability. Gust et al. (109) demonstrated widespread endothelial activation and increased BBB permeability, followed by leakage of systemic cytokines, including IFN-γ, into the cerebrospinal fluid. A recent study by Parker et al. (111), using single-cell RNA sequencing, revealed the expression of CD19 in brain mural cells surrounding the endothelium. This finding indicates the potential for CD19-targeted CAR T-cells to recognize CD19+ mural cells, leading to endothelial dysfunction and subsequent neurotoxicity. Anti-BCMA drugs, while associated with clinically significant incidences of ICANS, have also been linked to atypical neurotoxic events that appear distinct from ICANS in several clinical studies, including tremor and hemiparesis (32, 42). Although the mechanisms underlying these atypical neurotoxicities are not yet fully understood, the presence of BCMA expression in the basal ganglia and cerebellum could potentially explain the occurrence of atypical Parkinsonian symptoms associated with anti-BCMA drugs (112).

Both axicabtagene ciloleucel and tisagenlecleucel are anti-CD19 drugs but use different co-stimulatory domains. Axicabtagene ciloleucel uses CD28 as the co-stimulatory domain, and tisagenlecleucel uses 4-1BB as the co-stimulatory domain. In real-world studies, studies using axicabtagene ciloleucel with CD28 exhibited higher rates of both all-grade and high-grade ICANS than those using tisagenlecleucel with 4-1BB (both *P* < 0.001 for all-grade and high-grade ICANS). These differences in the incidence of ICANS are possibly related to its CD28 co-stimulation domain. In a previous study (113), CAR T-cells with CD28 co-stimulatory domains posed a greater risk for the development of ICANS. CD28 and 4-1BB operate via different mechanisms as co-stimulatory domains in CAR T-cells. The CD28-CD80/86 interaction enhances IL-2 activity, upregulates pro-survival gene transcription, and promotes Th1 cytokine production. CD28 co-stimulation is generally believed to preferentially expand CD4+ T cells. Conversely, 4-1BB, also known as CD137, is similar in its upregulation of anti-apoptotic proteins and IL-2, but it preferentially leads to CD8+ T cell expansion (114). Several studies have demonstrated that T cell expansion occurs more rapidly and with greater amplitude when stimulated by CD28 compared to 4-1BB *in vivo*. Salter et al. (115) observed significantly increased kinetics and intensity of T cell phosphorylation via mass spectrometry when stimulated by CD28/CD3ζ signaling compared to 4-1BB/CD3ζ signaling. Similarly, Sterner et al. (116) noted that CD28-stimulated CAR T-cells tend to differentiate into central memory T cells and rely on aerobic glycolysis, which may contribute to their faster onset and eventual exhaustion. These findings, altogether provide a biological basis for the higher incidence of ICANS observed in cohorts using axicabtagene ciloleucel with CD28 in our real-world study.

Imaging findings related to ICANS among patients undergoing CAR T-cell therapy have been variable and unspecific. The most common imaging findings associated with ICANS are cerebral vasogenic or cytotoxic edema, detected on T2-weighted and FLAIR as hyperintensities with or without diffusion restriction (1, 117). Reported locations of involvement include the bilateral thalami, brainstem, and splenium of the corpus callosum (113, 117). In a study by Santomasso et al. (113), brain MRI findings were normal in all five patients with grade 1 and 2 ICANS as well as in four of 14 patients with severe ICANS (113). Other abnormalities, including leptomeningeal enhancement and multifocal microhemorrhage, have also been reported (5). These imaging abnormalities may be explained by several potential pathophysiologic mechanisms: endothelial cell damage, disruption of the BBB, and systemic inflammation (117, 118).

The present study has, however, several limitations. First, the present meta-analysis was limited to cohort-level data, as patient-level data were not available. This restriction hindered us to assess individual patient-level risk factors associated with ICANS, such as disease burden, number of previous lines of treatment, age, and baseline cognitive function. Consequently, these patient-level factors, which might influence the development of ICANS, were not analyzed in our study. Second, there are a number of previous systemic reviews and articles about the adverse effects of CAR T-cell therapy. However, previous studies included only patients with specific diseases and analyzed only specific treatment agents. The advantage of this meta-analysis is that we included a total of 75 recent papers, covered the entire range of hematologic malignancies, and also performed multiple subgroups and multivariable meta-regression analyses. Third, despite our efforts to address heterogeneity through sensitivity analysis, subgroup analyses and meta-regression, substantial heterogeneity remained in some of the results. A relatively large sample size (75 studies) contributed to substantial heterogeneity in our results. Also, this heterogeneity reflects the diverse clinical settings and methodologies of the included studies, and underscores the need for cautious interpretation of our results. Lastly, the employed grading systems of neurotoxicity were heterogeneous, and we did not attempt to unify the schemes. There are, however, differences between these grading methods, and more sophisticated studies that correct for such differences are needed in the future. Overall, the results of our study should be interpreted with caution due to high levels of heterogeneity among the included studies.

## Conclusion

In conclusion, an overall incidence of ICANS among patients undergoing CAR T-cell therapy were 26.9% for all-grade and 10.5% for high-grade ICANS. Patients with anti-CD19 drugs had a significantly increased risk of developing high-grade ICANS compared to patients with anti-BCMA drugs. These results suggest that careful monitoring of ICANS should be considered for patients undergoing CAR T-cell therapy, particularly those treated with anti-CD19 drugs and those with leukemia.

## Data Availability

The original contributions presented in the study are included in the article/Supplementary material, further inquiries can be directed to the corresponding author.
