# Supplementary material for: Incidence of immune effector cell-associated neurotoxicity among patients treated with CAR T-cell therapy for hematologic malignancies: systematic review and meta-analysis
Source: Front Neurol. 2024 Oct 15;15:1392831. doi: 10.3389/fneur.2024.1392831 (PMC11518750; doi:10.3389/fneur.2024.1392831)
Supplement: Supplementary file 1 [file Data_Sheet_1.pdf]

Supplement

Literature search for analysis of real-world studies

For additional search for analysis of the incidence of ICANS in real-world clinical settings, 1390 articles were screened. Of the 1390 articles, 964 were excluded by an automation tool and by a review of the article titles. The complete texts of the remaining 426 articles were retrieved and 414 studies were further excluded after reviewing the full texts (320 articles were not studies of real-world clinical settings, 19 were not about CAR T-cell therapy, 14 did not discuss ICANS, 17 presented secondary analyses, 27 were summaries of other studies, 17 enrolled fewer than 100 patients). 12 studies were included in the analysis for real-world studies. These studies comprised a total of 3403 patients treated with CAR T-cell therapies for hematologic malignancy in real-world clinical setting.

Supplementary Table 1. PRISMA 2020 checklist.

Section and Topic	Item #	Checklist item	Location where item is reported
**TITLE**			
Title	1	Identify the report as a systematic review.	
**ABSTRACT**			
Abstract	2	See the PRISMA 2020 for Abstracts checklist.	
**INTRODUCTION**			
Rationale	3	Describe the rationale for the review in the context of existing knowledge.	
Objectives	4	Provide an explicit statement of the objective(s) or question(s) the review addresses.	
**METHODS**			
Eligibility criteria	5	Specify the inclusion and exclusion criteria for the review and how studies were grouped for the syntheses.	
Information sources	6	Specify all databases, registers, websites, organisations, reference lists and other sources searched or consulted to identify studies. Specify the date when each source was last searched or consulted.	
Search strategy	7	Present the full search strategies for all databases, registers and websites, including any filters and limits used.	
Selection process	8	Specify the methods used to decide whether a study met the inclusion criteria of the review, including how many reviewers screened each record and each report retrieved, whether they worked independently, and if applicable, details of automation tools used in the process.	
Data collection process	9	Specify the methods used to collect data from reports, including how many reviewers collected data from each report, whether they worked independently, any processes for obtaining or confirming data from study investigators, and if applicable, details of automation tools used in the process.	
Data items	10a	List and define all outcomes for which data were sought. Specify whether all results that were compatible with each outcome domain in each study were sought (e.g. for all measures, time points, analyses), and if not, the methods used to decide which results to collect.	
	10b	List and define all other variables for which data were sought (e.g. participant and intervention characteristics, funding sources). Describe any assumptions made about any missing or unclear information.	
Study risk of bias assessment	11	Specify the methods used to assess risk of bias in the included studies, including details of the tool(s) used, how many reviewers assessed each study and whether they worked independently, and if applicable, details of automation tools used in the process.	
Effect measures	12	Specify for each outcome the effect measure(s) (e.g. risk ratio, mean difference) used in the synthesis or presentation of results.	
Synthesis methods	13a	Describe the processes used to decide which studies were eligible for each synthesis (e.g. tabulating the study intervention characteristics and comparing against the planned groups for each synthesis (item #5)).	

Section and Topic	Item #	Checklist item	Location where item is reported
	13b	Describe any methods required to prepare the data for presentation or synthesis, such as handling of missing summary statistics, or data conversions.	
	13c	Describe any methods used to tabulate or visually display results of individual studies and syntheses.	
	13d	Describe any methods used to synthesize results and provide a rationale for the choice(s). If meta-analysis was performed, describe the model(s), method(s) to identify the presence and extent of statistical heterogeneity, and software package(s) used.	
	13e	Describe any methods used to explore possible causes of heterogeneity among study results (e.g. subgroup analysis, meta-regression).	
	13f	Describe any sensitivity analyses conducted to assess robustness of the synthesized results.	
Reporting bias assessment	14	Describe any methods used to assess risk of bias due to missing results in a synthesis (arising from reporting biases).	
Certainty assessment	15	Describe any methods used to assess certainty (or confidence) in the body of evidence for an outcome.	
**RESULTS**			
Study selection	16a	Describe the results of the search and selection process, from the number of records identified in the search to the number of studies included in the review, ideally using a flow diagram.	
	16b	Cite studies that might appear to meet the inclusion criteria, but which were excluded, and explain why they were excluded.	
Study characteristics	17	Cite each included study and present its characteristics.	
Risk of bias in studies	18	Present assessments of risk of bias for each included study.	
Results of individual studies	19	For all outcomes, present, for each study: (a) summary statistics for each group (where appropriate) and (b) an effect estimate and its precision (e.g. confidence/credible interval), ideally using structured tables or plots.	
Results of syntheses	20a	For each synthesis, briefly summarise the characteristics and risk of bias among contributing studies.	
	20b	Present results of all statistical syntheses conducted. If meta-analysis was done, present for each the summary estimate and its precision (e.g. confidence/credible interval) and measures of statistical heterogeneity. If comparing groups, describe the direction of the effect.	
	20c	Present results of all investigations of possible causes of heterogeneity among study results.	
	20d	Present results of all sensitivity analyses conducted to assess the robustness of the synthesized results.	
Reporting biases	21	Present assessments of risk of bias due to missing results (arising from reporting biases) for each synthesis assessed.	
Certainty of evidence	22	Present assessments of certainty (or confidence) in the body of evidence for each outcome assessed.	

Section and Topic	Item #	Checklist item	Location where item is reported
**DISCUSSION**			
Discussion	23a	Provide a general interpretation of the results in the context of other evidence.	
	23b	Discuss any limitations of the evidence included in the review.	
	23c	Discuss any limitations of the review processes used.	
	23d	Discuss implications of the results for practice, policy, and future research.	
**OTHER INFORMATION**			
Registration and protocol	24a	Provide registration information for the review, including register name and registration number, or state that the review was not registered.	
	24b	Indicate where the review protocol can be accessed, or state that a protocol was not prepared.	
	24c	Describe and explain any amendments to information provided at registration or in the protocol.	
Support	25	Describe sources of financial or non-financial support for the review, and the role of the funders or sponsors in the review.	
Competing interests	26	Declare any competing interests of review authors.	
Availability of data, code and other materials	27	Report which of the following are publicly available and where they can be found: template data collection forms; data extracted from included studies; data used for all analyses; analytic code; any other materials used in the review.	

Supplementary Table 2. Search terms for the meta-analysis in PUBMED.

No.	Search Query	Results
#1	"Receptors, Chimeric Antigen"[Mesh]	3,639
#2	"Chimeric antigen receptor*"[TW] OR CAR[TW]	36,317
#3	#1 OR #2	36,485
#4	"Receptors, Antigen, T-Cell"[Mesh]	45,043
#5	T-Cell*[TW]	441,924
#6	#4 OR #5	443,738
#7	"Immunotherapy, Adoptive"[Mesh]	12,960
#8	Immunotherap*[TW] OR Therap*[TW] OR treatment*[TW]	9,801,448
#9	#7 OR #8	9,801,448
#10	#3 AND #6 AND #9	9,957
#11	"CAR T-cell*"[TW] AND (therap*[TW] OR treatment*[TW])	6,413
#12	("CAR therap*"[TW] OR "CAR treatment*"[TW]) AND "T-cell*"[TW]	176
#13	"axicabtagene ciloleucel" [Supplementary Concept] OR axicabtagene[TW]	375
#14	"brexucabtagene autoleucel" [Supplementary Concept] OR "brexucabtagene autoleucel"[TW]	53
#15	"idecabtagene vicleucel" [Supplementary Concept] OR "idecabtagene vicleucel"[TW]	59
#16	"tisagenlecleucel" [Supplementary Concept] OR "tisagenlecleucel" [TW]	457
#17	"Lisocabtagene maraleucel"[TW]	71
#18	"ciltacabtagene autoleucel"[TW]	38
#19	#10 OR #11 OR #12 OR #13 OR #14 OR #15 OR #16 OR #17 OR #18	10,064
#20	"Neurotoxicity Syndromes"[Mesh] OR "Nervous System Diseases"[Mesh:Noexp]	78,471
#21	Neurotoxic*[TW] OR Neurotoxin*[TW] OR ICANS[TW]	87,093

#22	(neurol*[TW] OR "Nervous System*"[TW] OR Encephalopat*[TW]) AND (Toxic*[TW] OR event*[TW] OR complicat*[TW] OR disease*[TW] OR Disorder*[TW] OR Poisoning*[TW])	648,184
#23	#20 OR #21 OR #22	733,744
#24	#19 AND #23	1,001
#25	#24 AND ("Clinical Trial"[ptyp] OR trial*[TW] OR "clinical trial*"[TW] OR random*[TW] OR RCT[TW] OR RCTs[TW] OR Placebo*[TW] OR Blind*[TW] OR "Controlled Clinical"[TW])	477
#26	#25 NOT (animals[Mesh:noexp] NOT (animals[Mesh:noexp] AND humans[Mesh]))	477
#27	#26 AND English[lang] AND ("1950/01/01"[PDAT] : "2022/05/28"[PDAT])	385
#28	#27 NOT ("Review"[ptyp] OR "Systematic Review"[ptyp] OR "Meta-Analysis"[ptyp] OR Review*[TI] OR Meta-Analys*[TI] OR "Systematic Literature*"[TI] OR Autobiography[ptyp] OR Bibliography[ptyp] OR Biography[ptyp] OR pubmed books[filter] OR Comment[ptyp] OR Dataset[ptyp] OR Dictionary[ptyp] OR Editorial[ptyp] OR Electronic Supplementary Materials[ptyp] OR Interview[ptyp] OR Legislation[ptyp] OR News[ptyp] OR Newspaper Article[ptyp] OR Retracted Publication[sb] OR Retraction of Publication[sb] OR Technical Report[ptyp] OR Letter[ptyp])	222

Supplementary Table 3. Search terms for the meta-analysis in EMBASE.

No.	Search Query	Results
#1	'chimeric antigen receptor T-cell immunotherapy'/exp	7,510
#2	'chimeric antigen receptor'/exp AND 'lymphocyte antigen receptor'/exp AND 'adoptive immunotherapy'/exp	95
#3	(('Chimeric antigen receptor*' OR CAR) NEAR/6 'T-Cell*' NEAR/6 (Immunotherap* OR Therap* OR treatment*)):ab,ti,kw	12,115
#4	('CAR-T' NEAR/6 'Cell*' NEAR/6 (Immunotherap* OR Therap* OR treatment*)):ab,ti,kw	10,518
#5	'axicabtagene ciloleucel'/exp OR axicabtagene:ab,ti,kw	1,891
#6	'brexucabtagene autoleucel'/exp OR 'brexucabtagene autoleucel':ab,ti,kw	312
#7	'idecabtagene vicleucel'/exp OR 'idecabtagene vicleucel':ab,ti,kw	360
#8	'tisagenlecleucel T'/exp OR 'tisagenlecleucel':ab,ti,kw	2,301
#9	'lisocabtagene maraleucel'/exp OR 'Lisocabtagene maraleucel':ab,ti,kw	464
#10	'ciltacabtagene autoleucel'/exp OR 'ciltacabtagene autoleucel':ab,ti,kw	231
#11	#1 OR #2 OR #3 OR #4 OR #5 OR #6 OR #7 OR #8 OR #9 OR #10	15,962
#12	'neurotoxicity'/exp OR 'toxicity and intoxication'/exp	1,108,907
#13	(Neurotoxic* OR Neurotoxin* OR ICANS):ab,ti,kw	103,087
#14	((neurol* OR 'Nervous System*' OR Encephalopat*) NEAR/6 (Toxic* OR event* OR complicat* OR disease* OR Disorder* OR Poisoning*)):ab,ti,kw	240,624
#15	#12 OR #13 OR #14	1,372,807
#16	#11 AND #15	3,798
#17	#16 AND ('clinical trial'/exp OR [randomized controlled trial]/lim OR 'controlled clinical trial'/de OR trial* OR 'clinical trial*' OR random* OR RCT OR RCTs OR Placebo* OR Blind* OR 'Controlled Clinical')	2,206
#18	#17 NOT ('animal'/de NOT ('animal'/de AND 'human'/exp))	2,206
#19	#18 AND [english]/lim AND [01-01-1966]/sd NOT [29-05-2022]/sd	1,806
#20	#19 AND ([article]/lim OR [article in press]/lim)	290

Supplementary Table 4. Search terms for the meta-analysis in Web of Science.

No.	Search Query	Results
#1	TS=(("Chimeric antigen receptor*" OR CAR) NEAR/6 "T-Cell*" NEAR/6 (Immunotherap* OR Therap* OR treatment*))	7,176
#2	TS=("CAR-T" NEAR/6 "Cell*" NEAR/6 (Immunotherap* OR Therap* OR treatment*))	5,651
#3	TS=(axicabtagene OR "brexucabtagene autoleucel" OR "idecabtagene vicleucel" OR "tisagenlecleucel" OR "Lisocabtagene maraleucel" OR "ciltacabtagene autoleucel")	1,164
#4	#1 OR #2 OR #3	7,847
#5	TS=(Neurotoxic* OR Neurotoxin* OR ICANS)	97,415
#6	TS=((neurol* OR "Nervous System*" OR Encephalopat*) NEAR/6 (Toxic* OR event* OR complicat* OR disease* OR Disorder* OR Poisoning*))	147,735
#7	#5 OR #6	239,331
#8	#4 AND #7	776
#9	#8 AND TS=(trial* OR "clinical trial*" OR random* OR RCT OR RCTs OR Placebo* OR Blind* OR "Controlled Clinical")	311
#10	#9 Editions: WOS.SCI Timespan: 1983-01-01 to 2022-05-28	264
#11	#10 AND Article (Document Types) and English (Languages)	143

Supplementary Table 5. Risk of bias assessment of the included studies.

	Risk of Bias					
	Randomization	allocation concealment	blinding of participants and personnel	blinding of outcome assessment	incomplete outcome data	selective outcome data
Abramson JS et al 2020 (1)	High	High	High	Low	Low	Unclear
An F et al 2020 (2)	High	High	High	Low	Low	Unclear
Baumeister SH et al 2019 (3)	Unclear	Unclear	Unclear	Unclear	Low	Unclear
Benjamin R et al 2020 (4)	High	High	High	Low	Low	Unclear
Berdeja JG et al 2021 (5)	High	High	High	Low	Low	Unclear
Brudno JN et al 2018 (6)	Unclear	Unclear	Unclear	Unclear	Low	Unclear
Brudno JN et al 2020 (7)	High	High	High	Low	Low	Low
Caimi PF et al 2021 (8)	Unclear	Unclear	Unclear	Unclear	Low	Unclear
Cao J et al 2018 (9)	Unclear	Unclear	Unclear	Unclear	Low	Unclear
Cohen AD et al 2019 (10)	Unclear	High	High	Low	Low	Unclear
Cordoba S et al 2021 (11)	High	High	High	Low	Low	Unclear
Cornell RF et al 2021 (12)	Unclear	High	High	Low	Low	Unclear
Curran KJ et al 2019 (13)	Unclear	Unclear	Unclear	Unclear	Low	Unclear
Fan L et al 2022 (14)	Unclear	High	High	Low	Low	Unclear
Fowler NH et al 2022 (15)	High	High	High	Low	Low	Low
Frey NV et al 2020 (16)	High	Unclear	Unclear	Unclear	Low	Unclear
Gardner RA et al 2017 (17)	Unclear	Unclear	Unclear	Unclear	Low	Unclear
Geyer MB et al 2019 (18)	High	High	High	Low	High	Unclear
Heng G et al 2020 (19)	High	High	High	Low	Low	Unclear
Jacobson CA et al 2022 (20)	High	High	High	Low	Low	Low
Jacoby E et al 2018 (21)	Unclear	Unclear	Unclear	Unclear	Low	Unclear
Kato K et al 2022 (22)	High	High	High	Low	Low	Unclear
Kochenderfer JN et al 2017 (23)	Unclear	Unclear	Unclear	Unclear	Low	Unclear
Lamure S et al 2021 (24)	Unclear	Unclear	Unclear	Unclear	Low	Unclear

Li C et al 2021 (25)	High	Unclear	Unclear	Unclear	Low	Unclear
Liu H et al 2021 (26)	High	High	High	Low	Low	Unclear
Liu Y et al 2022 (27)	High	Unclear	Unclear	Unclear	Low	Unclear
Locke FL et al 2022 (28)	Low	Low	Low	Low	Low	Low
Ma F et al 2019 (29)	Unclear	Unclear	Unclear	Unclear	Low	Unclear
Magnani CF et al 2020 (30)	Unclear	Unclear	Unclear	Unclear	Low	Unclear
Makita S et al 2022 (31)	High	High	High	Low	Low	Unclear
Maude SL et al 2018 (32)	High	High	High	Low	Low	Low
Mei H et al 2021 (33)	High	High	High	Low	Low	Unclear
Munshi NC et al 2021 (34)	High	High	High	Low	Low	Low
Myer RM et al 2021 (35)	High	Unclear	Unclear	Unclear	Low	Unclear
Neelapu SS et al 2017 (36)	High	High	High	Low	Low	Low
Neelapu SS et al 2022 (37)	High	High	High	Low	Low	Low
Ortíz-Maldonado V et al 2021 (25)	High	High	High	Low	High	Unclear
Pan J et al 2019 (38)	High	Unclear	Unclear	Unclear	Low	Unclear
Pan J et al 2021 (39)	High	Unclear	Unclear	Unclear	Low	Unclear
Park JH et al 2018 (40)	Unclear	Unclear	Unclear	Unclear	Low	Unclear
Raje N et al 2019 (41)	High	High	High	Low	Low	Low
Ramos CA et al 2018 (42)	Unclear	Unclear	Unclear	Unclear	Low	Unclear
Ramos CA et al 2020 (43)	High	Unclear	Unclear	Unclear	Low	Low
Roddie C et al 2021 (44)	High	High	High	Low	Low	Low
Sang W et al 2020 (45)	Unclear	Unclear	Unclear	Unclear	Low	Unclear
Sauter CS et al 2019 (46)	Unclear	Unclear	Unclear	Unclear	Low	Unclear
Schuster SJ et al 2019 (47)	High	High	High	Low	Low	Low
Shah BD et al 2021 (48)	High	High	High	Low	Low	Unclear
Shah BD et al 2021 (49)	High	High	High	Low	Low	Low
Shah NN et al 2020 (50)	Unclear	Unclear	Unclear	Unclear	Low	Unclear
Shah NN et al 2020 (51)	High	High	High	Low	Low	Unclear
Shalabi H et al 2022 (52)	Unclear	Unclear	Unclear	Unclear	Low	Unclear
Shi X et al 2022 (53)	High	High	High	Low	Low	Unclear
Siddiqi T et al 2022 (54)	Unclear	High	High	Low	Low	Unclear

Singh H et al 2022 (55)	High	High	High	Low	Low	Unclear
Spiegel JY et al 2021 (56)	High	High	High	Low	Low	Low
Talleur A et al 2022 (57)	Unclear	Unclear	Unclear	Unclear	Low	Unclear
Tong C et al 2020 (58)	High	High	High	Low	Low	Unclear
Turtle CJ et al 2016 (59)	Unclear	High	High	Low	Low	Unclear
Wang D et al 2021 (60)	High	High	High	Low	Low	Unclear
Wang J et al 2022 (61)	Unclear	Unclear	Unclear	Unclear	Low	Unclear
Wang M et al 2020 (62)	High	High	High	Low	Low	Low
Wang Y et al 2022 (63)	High	High	High	Low	Low	Unclear
Wei G et al 2021 (64)	Unclear	High	High	Low	Low	Unclear
Wu J et al 2021 (65)	High	High	High	Low	Low	Unclear
Yan ZX et al 2019 (66)	High	High	High	Low	Low	Unclear
Ying Z et al 2019 (67)	High	High	High	Low	Low	Unclear
Ying Z et al 2021 (68)	Unclear	High	High	Low	Low	Unclear
Ying Z et al 2022 (69)	Unclear	Unclear	Unclear	Unclear	Low	Unclear
Zhang H et al 2022 (70)	High	High	High	Low	Low	Unclear
Zhang X et al 2020 (71)	Unclear	Unclear	Unclear	Unclear	Low	Unclear
Zhang Y et al 2021 (72)	High	Unclear	Unclear	Unclear	Low	Unclear
Zhao WH et al 2018 (73)	High	High	High	Low	Low	Unclear
Zhou X et al 2020 (74)	High	High	High	Low	Low	Unclear

Supplementary Table 6. Characteristics of all eligible trial cohorts

Study	Agents	N	ICANS			ICANS grading method	Dose of CAR T cells agents	Ratio of CD4: CD8
			All grade	High grade	Grade 5			
Abramson JS et al 2020 (1)	anti-CD19	269	80	27	0	CTCAE	50 – 150×10^6/kg	1:1
An F et al 2020 (2)	anti-CD19	47	3	1	0	CTCAE	1 – 5×10^6/kg	NA
Baumeister SH et al 2019 (3)	NKG2D-CAR	12	0	0	0	CTCAE	1– 30×10^6/kg	Median 2.05
Benjamin R et al 2020 (4)	anti-CD19	21	8	0	0	CTCAE	1·1 – 2·3×10^6/kg (child)<br>6 – 180×10^6/kg (adult)	2.1 and 1.9
Berdeja JG et al 2021 (5)	anti-BCMA	97	20	9	1	CTCAE (phase 1b)<br>ASTCT (phase 2)	0.5 – 1×10^6/kg	NA
Brudno JN et al 2018 (6)	anti-BCMA	16	1	1	0	CTCAE	0.3 – 3×10^6/kg	NA
Brudno JN et al 2020 (7)	anti-CD19	20	4	1	0	CTCAE	0.66 – 6×10^6/kg	NA
Caimi PF et al 2021 (8)	anti-CD19	20	5	1	0	CTCAE	0.5 – 2×10^6/kg	Median 66:34
Cao J et al 2018 (9)	anti-CD19	18	1	0	0	NA	1×10^6/kg	NA
Cohen AD et al 2019 (10)	anti-BCMA	25	8	3	0	CTCAE	0.1 – 5×10^8	Median 1.7
Cordoba S et al 2021 (11)	anti-CD19 &<br>anti-CD22	15	4	0	0	ASBMT	1 – 3×10^6/kg	1:1
Cornell RF et al 2021 (12)	anti-BCMA	14	3	0	0	NA	3×10^7 – 1×10^9	NA
Curran KJ et al 2019 (13)	anti-CD19	25	18	7	NA	CTCAE	1 – 3×10^6/kg	NA
Fan L et al 2022 (14)	anti-CD19	10	0	0	0	CTCAE	0.8 – 5×10^6/kg	NA
Fowler NH et al 2022 (15)	anti-CD19	97	36	3	0	CTCAE	0.6 – 6×10^8/kg	NA
Frey NV et al 2020 (16)	anti-CD19	38	13	6	0	ASTCT	0.5 – 5×10^8/kg	NA
Gardner RA et al 2017 (17)	anti-CD19	45	21	9	0	NA	0.5 – 10×10^6/kg	NA
Geyer MB et al 2019 (18)	anti-CD19	20	9	2	0	CTCAE	1 – 3×10^7/kg	Median 1.9:1
Heng G et al 2020 (19)	anti-CD19	10	4	0	NA	CTCAE	0.23 – 41.67×10^6/kg	Median 0.70

Jacobson CA et al 2022 (20)	anti-CD19	148	87	28	0	CTCAE	2×10^6/kg	NA
Jacoby E et al 2018 (21)	anti-CD19	20	11	0	0	CTCAE	1×10^6/kg	32.9:61.3
Kato K et al 2022 (22)	anti-CD19	16	0	0	0	CTCAE	2×10^6/kg	NA
Kochenderfer JN et al 2017 (23)	anti-CD19	22	22	12	0	NA	$1 – 6\times10^6$/kg	NA
Lamure S et al 2021 (24)	anti-CD19	60	23	7	0	ASTCT	NA	NA
Li C et al 2021 (25)	anti-BCMA	30	1	0	0	ASTCT	Median 11.2×10^6/kg	NA
Liu H et al 2020 (26)	anti-CD19	17	17	0	0	ASTCT	$0.5 – 4 \times10^6$/kg	NA
Liu Y et al 2022 (27)	anti-CD19 & anti-CD22	23	8	5	0	ASTCT	$0.1 – 5\times10^6$/kg	NA
Locke FL et al 2022 (75)	anti-CD19	170	102	36	0	CTCAE	2×10^6/kg	NA
Ma F et al 2019 (29)	anti-CD19	10	6	6	0	CTCAE	$0.3 – 1.58\times10^6$/kg	0.7
Magnani CF et al 2020 (30)	anti-CD19	13	0	0	0	CTCAE	$0.53 – 2.2 \times 10^6$/kg	NA
Makita S et al 2022 (31)	anti-CD19	10	1	0	0	CTCAE	100×10^6/kg (total)	1:1
Maude SL et al 2018 (32)	anti-CD19	75	30	10	0	CTCAE	$0.2 – 250\times10^6$/kg	1.7
Mei H et al 2021 (33)	anti-BCMA & anti-CD38	23	0	0	0	CTCAE	$0.5 – 1\times10^6$/kg	39.5: 58.5
Munshi NC et al 2021 (34)	anti-BCMA	128	23	4	0	CTCAE	$150 – 450\times10^6$/kg	NA
Myers RM et al 2021 (35)	anti-CD19	74	29	3	0	CTCAE	$0.3 – 30 \times10^6$/kg	NA
Neelapu SS et al 2017 (36)	anti-CD19	101	65	28	0	CTCAE	2×10^6/kg	NA
Neelapu SS et al 2022 (37)	anti-CD19	40	29	9	0	CTCAE	2×10^6/kg	1:1
Ortiz-Maldonado V et al 2021 (25)	anti-CD19	47	0	1	NA	CTCAE	$0.4 – 5\times10^6$/kg	100:85
Pan J et al 2019 (38)	anti-CD22	34	6	0	0	CTCAE	Median 7.5×10^6/kg	NA
Pan J et al 2021 (39)	anti-CD7	20	3	0	0	ASTCT	1×10^6/kg	33:61
Park JH et al 2018 (40)	anti-CD19	53	23	22	0	CTCAE	$1 – 3\times10^6$/kg	NA
Raje N et al 2019 (41)	anti-BCMA	33	14	1	0	CTCAE	$50 – 800\times10^6$/kg	Median 85:13
Ramos CA 2018 (42)	anti-CD19	16	1	1	0	CTCAE	$1 – 20 \times10^6$/kg	NA
Ramos CA et al 2020 (43)	anti-CD30	42	0	0	0	CTCAE	$2 – 20\times10^7$/kg	NA

Roddie C et al 2021 (44)	anti-CD19	20	4	3	0	ASTCT	10 – 100×10^{6}/kg	Mean 3.56
Sang W et al 2020 (45)	anti-CD19 & anti-CD20	21	5	2	0	CTCAE	Median 0.71×10^{6}/kg	AVG 0.83
Sauter CS et al 2019 (46)	anti-CD19	15	10	9	0	CTCAE	0.8 – 5×10^{6}/kg	NA
Schuster SJ et al 2019 (47)	anti-CD19	111	23	13	0	CTCAE	2.5 – 25×10^{5}/kg	3.1
Shah BD et al 2021 (48)	anti-CD19	45	35	17	0	CTCAE	Median 0.5×10^{6}/kg	NA
Shah BD et al 2021(49)	anti-CD19	55	33	14	1	CTCAE	2×10^{6}/kg	NA
Shah NN et al 2020 (50)	anti-CD22	58	19	1	0	CTCAE	0.4 – 5×10^{6}/kg	NA
Shah NN et al 2020 (51)	anti-CD19 & anti-CD20	22	7	3	0	CTCAE	0.1 – 1×10^{6}/kg	NA
Shalabi H et al 2022 (52)	anti-CD19 & anti-CD28	52	11	4	0	CTCAE	Median 8.258×10^{8}/kg	NA
Shi X et al 2022 (53)	anti-CD19 & anti-BCMA	10	0	0	0	CTCAE	Median 1×10^{6}/kg (anti-CD19)<br>Median 1×10^{6}/kg (anti-CD20)	NA
Siddiqi T et al 2022 (54)	anti-CD19	23	9	5	0	CTCAE	3 – 100×10^{7}/kg	NA
Singh H et al 2022 (55)	anti-CD19	14	0	0	0	CTCAE	0.1 – 1000×10^{6}/kg	NA
Spiegel JY et al 2021 (56)	anti-CD19 & anti-CD22	38	14	4	0	ASTCT	1 – 10 ×10^{6}/kg	NA
Talleur A et al 2022 (57)	anti-CD19	12	3	1	0	CTCAE & ASTCT	5 – 10×10^{7}/kg	1
Tong C et al 2020 (58)	anti-CD19 & anti-CD 20	61	6	0	0	CTCAE	0.5 – 6×10^{6}/kg	NA
Turtle CJ et al 2016 (59)	anti-CD19	30	15	15	1	CTCAE	2.5 – 10×10^{7}/kg	NA
Wang D et al 2021 (60)	anti-BCMA	18	0	0	0	CTCAE	1.5 – 4.5×10^{8}/kg	NA
Wang J et al 2022 (61)	anti-CD19	18	2	2	0	ASTCT	0.5 – 5 ×10^{6}/kg	NA
Wang M et al 2020 (62)	anti-CD19	68	43	21	0	CTCAE	1 – 15×10^{6}/kg	NA
Wang Y et al 2022 (63)	anti-CD19 & anti-BCMA	62	7	2	0	CTCAE	1×10^{6}/kg	NA
Wei G et al 2021 (64)	anti-CD19 & anti-CD22	16	0	0	0	CTCAE	2×10^{6}/kg	NA

Wu J et al 2021 (65)	anti-CD19 & anti-CD22	13	3	1	0	ASTCT	0.5 – 2×10^6/kg Unknown (2)	1.94
Yan ZX et al 2019 (66)	anti-CD19	10	1	1	0	CTCAE	1 – 3×10^7/kg	NA
Ying Z et al 2019 (67)	anti-CD19	25	0	0	0	CTCAE	1×10^8/kg	1
Ying Z et al 2021 (68)	anti-CD19	59	12	3	0	CTCAE	0.75×10^6/kg	NA
Ying Z et al 2022 (69)	anti-CD19	22	1	1	0	ASTCT	0.5 – 3×10^6/kg	NA
Zhang H et al 2022 (70)	anti-BCMA & anti-CD38	22	3	0	0	ASTCT	Median 2.06×10^8/kg	NA
Zhang X et al 2020 (71)	anti-CD19	110	23	15	0	ASTCT	1–3×10^6/kg	NA
Zhang Y et al 2021 (72)	anti-CD19 & anti-CD22	32	5	4	0	ASBMT	2×10^6/kg	NA
Zhao WH et al 2018 (73)	anti-BCMA	57	1	0	0	CTCAE	1–1.5×10^8/kg	NA
Zhou X et al 2020 (74)	anti-CD19	21	1	1	0	MDACC scale	NA	NA

Note. NA, not available; BCMA, B-cell maturation antigen; CAR, chimeric antigen receptor; ICANS, immune effector cell-associated neurotoxicity syndrome; CTCAE, National Cancer Institute Common Terminology Criteria for Adverse Events v4.03; ASTCT, American Society for Transplantation and Cellular Therapy scales; MDACC, MD Anderson Cancer Center Scale

Supplementary Table 7. Characteristics of all-grade ICANS in all eligible trial cohorts and real-world studies

Study	ICANS onset, median (range or IQR), days	ICANS duration, median (range or IQR), days	Treatment outcomes
Abramson JS et al 2020 (1)	9 (range, 1 – 66)	11 (range, 1 – 86)	NA
An F et al 2020 (2)	NA	NA	NA
Baumeister SH et al 2019 (3)	NA	NA	NA
Benjamin R et al 2020 (4)	9 (IQR, 5 – 14)	3 (IQR, 2 – 4)	All resolved without treatment
Berdeja JG et al 2021 (5)	8 (IQR, 6 – 8)	4 (IQR, 3 – 6.5)	All resolved with steroid, tocilizumab, or anakinra
Brudno JN et al 2018 (6)	NA	NA	NA
Brudno JN et al 2020 (7)	NA	1	All resolved with steroid
Caimi PF et al 2021 (8)	7 (range, 5 – 9)	NA	All resolved with steroid, or anakinra
Cao J et al 2018 (9)	14	5	All resolved with mannitol
Cohen AD et al 2019 (10)	NA	NA	All resolved with steroid + cyclophosphamide
Cordoba S et al 2021 (11)	5.5 (range, 3 – 9)	5 (range, 1 – 10)	75% (3/4) resolved
Cornell RF et al 2021 (12)	NA	NA	NA
Curran KJ et al 2019 (13)	NA	NA	83% (15/18) resolved with supportive treatment or steroid
Fan L et al 2022 (14)	NA	NA	NA
Fowler NH et al 2022 (15)	9 (IQR, 5 – 35)	2 (IQR, 1 – 4)	All resolved
Frey NV et al 2020 (16)	NA	NA	NA
Gardner RA et al 2017 (17)	NA	NA	All resolved
Geyer MB et al 2019 (18)	2 (range, 1 – 11)	1 (range, 1 – 61)	All resolved with steroid
Heng G et al 2020 (19)	NA	NA	All resolved with steroid, tocilizumab, or mannitol
Jacobson CA et al 2022 (20)	7 (IQR, 6 – 10) for FL<br>7 (IQR, 6 – 11) for MZL	14 (IQR, 5 – 43) for FL<br>10 (IQR, 5 – 28) for MZL	95% (83/87) resolved with steroid or tocilizumab
Jacoby E et al 2018 (21)	NA	2.5 (range, 0 – 11)	All resolved with steroid

Kato K et al 2022 (22)	NA	NA	NA
Kochenderfer JN et al 2017 (23)	NA	NA	All resolved with steroid or tocilizumab
Lamure S et al 2021 (24)	NA	NA	NA
Li C et al 2021 (25)	3	2	All resolved with steroid or mannitol
Liu H et al 2020 (26)	NA	NA	NA
Liu Y et al 2022 (27)	6.4 (range, 5 – 10)	5.5 (range, 3 – 10)	All resolved with steroid
Locke FL et al 2022 (75)	7	9	99% (101/102) resolved
Ma F et al 2019 (29)	NA	NA	NA
Magnani CF et al 2020 (30)	NA	NA	NA
Makita S et al 2022 (31)	4	3	All resolved without treatment
Maude SL et al 2018 (32)	NA	NA	87% (26/30) resolved with supportive treatment
Mei H et al 2021 (33)	NA	NA	NA
Munshi NC et al 2021 (34)	2 (range, 1 – 10)	3 (range, 1 – 26)	NA
Myers RM et al 2021 (35)	NA	NA	All resolved
Neelapu SS et al 2017 (36)	5 (range, 1 – 17)	12	94% (61/65) resolved with tocilizumab or steroid
Neelapu SS et al 2022 (37)	9 (range, 2 – 44)	7	97% (28/29) resolved with tocilizumab or steroid
Ortiz-Maldonado V et al 2021 (25)	NA	NA	NA
Pan J et al 2019 (38)	8 (range, 1 – 17)	7	Treated with mannitol, furosemide, or steroid
Pan J et al 2021 (39)	1 (range, 1 – 4)	3 (range, 2 – 4)	NA
Park JH et al 2018 (40)	NA	NA	NA
Raje N et al 2019 (41)	5 (range, 3 – 11)	8 (range, 1 – 251)	Treated with steroid
Ramos CA 2018 (42)	NA	NA	All resolved with tocilizumab + steroid
Ramos CA et al 2020 (43)	NA	NA	NA
Roddie C et al 2021 (44)	22 (range, 14 – 41)	1.5 (range, 1 – 8)	All resolved with steroid
Sang W et al 2020 (45)	NA	5 (range, 3 – 8)	NA
Sauter CS et al 2019 (46)	5 (range, 1 – 6)	9.5 (range, 2 – 20)	All resolved with tocilizumab, steroid, or without treatment
Schuster SJ et al 2019 (47)	6 (range, 1 – 17)	14	Majority resolved with supportive treatment

Shah BD et al 2021 (48)	9 (IQR, 7 – 11)	7 (IQR, 4 – 19)	88% (29/33) resolved with tocilizumab, steroid, or vasopressor
Shah BD et al 2021(49)	6 (IQR, 3 – 8)	12 (IQR, 4 – 26)	89% (31/35) resolved with tocilizumab or steroid
Shah NN et al 2020 (50)	6 (range, 0 – 13)	NA	All resolved with tocilizumab or steroid
Shah NN et al 2020 (51)	NA	NA	95% (18/19) resolved
Shalabi H et al 2022 (52)	9 (range, 3 – 11)	5 (range, 1 – 15)	All resolved with tocilizumab or steroid
Shi X et al 2022 (53)	NA	NA	NA
Siddiqi T et al 2022 (54)	4 (range, 2 – 21)	20.5 (range, 6 – 50)	All resolved with tocilizumab or steroid
Singh H et al 2022 (55)	NA	NA	NA
Spiegel JY et al 2021 (56)	5 (range, 3 – 9)	4 (range, 1 – 11)	All resolved with tocilizumab or steroid
Talleur A et al 2022 (57)	6 (range, 5 – 7)	NA	All resolved with siltuximab
Tong C et al 2020 (58)	NA	NA	NA
Turtle CJ et al 2016 (59)	NA	NA	NA
Wang D et al 2021 (60)	NA	NA	NA
Wang J et al 2022 (61)	NA	NA	All resolved with steroid
Wang M et al 2020 (62)	7 (range, 1 – 32)	12	86% (37/43) resolved
Wang Y et al 2022 (63)	Within 3 months	NA	Treated with tocilizumab or steroid
Wei G et al 2021 (64)	NA	NA	NA
Wu J et al 2021 (65)	NA	NA	All resolved with antibiotics with or without steroid
Yan ZX et al 2019 (66)	NA	NA	100% (1/1) with ongoing symptoms
Ying Z et al 2019 (67)	NA	NA	NA
Ying Z et al 2021 (68)	8.5	12.5	All resolved with tocilizumab or steroid
Ying Z et al 2022 (69)	NA	NA	All resolved with tocilizumab or steroid
Zhang H et al 2022 (70)	NA	NA	All resolved with supportive treatment
Zhang X et al 2020 (71)	NA	NA	All resolved with tocilizumab with or without steroids
Zhang Y et al 2021 (72)	11.5 (range, 8 – 20)	NA	80% (4/5) resolved with steroid and supportive treatment
Zhao WH et al 2018 (73)	NA	NA	All resolved with diazepam
Zhou X et al 2020 (74)	33	NA	All resolved with steroid
Bastos-Oreiro M et al 2022 (76)	NA	NA	NA

Bethge WA et al 2022 (77)	NA	NA	NA
Gauthier J et al 2022 (78)	NA	6 (IQR, 3 – 14; range, 1 – 101) for axi-cel<br>5 (IQR, 2 – 7; range, 1 – 27) for tisa-cel	Treated with tocilizumab or steroid
Ghilardi G et al 2022 (79)	NA	NA	Treated with tocilizumab or steroid
Kuhnl A et al 2022 (80)	NA	NA	Treated with tocilizumab or steroid
Kwon M et al 2022 (81)	7 (range, 2 – 65) for axi-cel<br>6 (range, 2 – 35) for tisa-cel	4 (range, 1 – 44) for axi-cel<br>7 (range, 1 – 83) for tisa-cel	Treated with tocilizumab, steroid, anakinra, or siltuximab
Nastoupil LJ et al 2020 (82)	NA	NA	99% (188/189) resolved with tocilizumab or steroid
Pasquini MC et al 2020 (83)	7 (range, 1 – 80) for ALL<br>8 (range, 2 – 33) for NHL	7 (range, 1 – 94) for ALL<br>6.5 (range, 1 – 50) for NHL	Treated with steroid
Riedell PA et al 2022 (84)	6 (IQR, 4 – 8) for axi-cel<br>4 (IQR, 3 – 7) for tisa-cel	7 (IQR, 4 – 11) for axi-cel<br>4 (IQR, 3 – 9) for tisa-cel	Treated with tocilizumab or steroid
Rubin DB et al 2020 (85)	6 (IQR, 5 – 8)	NA	Treated with tocilizumab
Strati P et al 2020 (86)	5 (range, 0 – 25)	6 (range, 0 – 52)	99% (67/68) resolved with antiseizure treatment, steroid, or tocilizumab
Zettler ME et al 2021 (87)	NA	NA	NA

Note. NA, not available; ICANS, immune effector cell-associated neurotoxicity syndrome; IQR, interquartile range; FL, follicular lymphoma; MZL, marginal zone lymphoma; ALL, acute lymphoblastic leukemia; NHL, non-Hodgkin lymphoma

Supplementary Figure 1. Risk of bias assessment of the included studies.

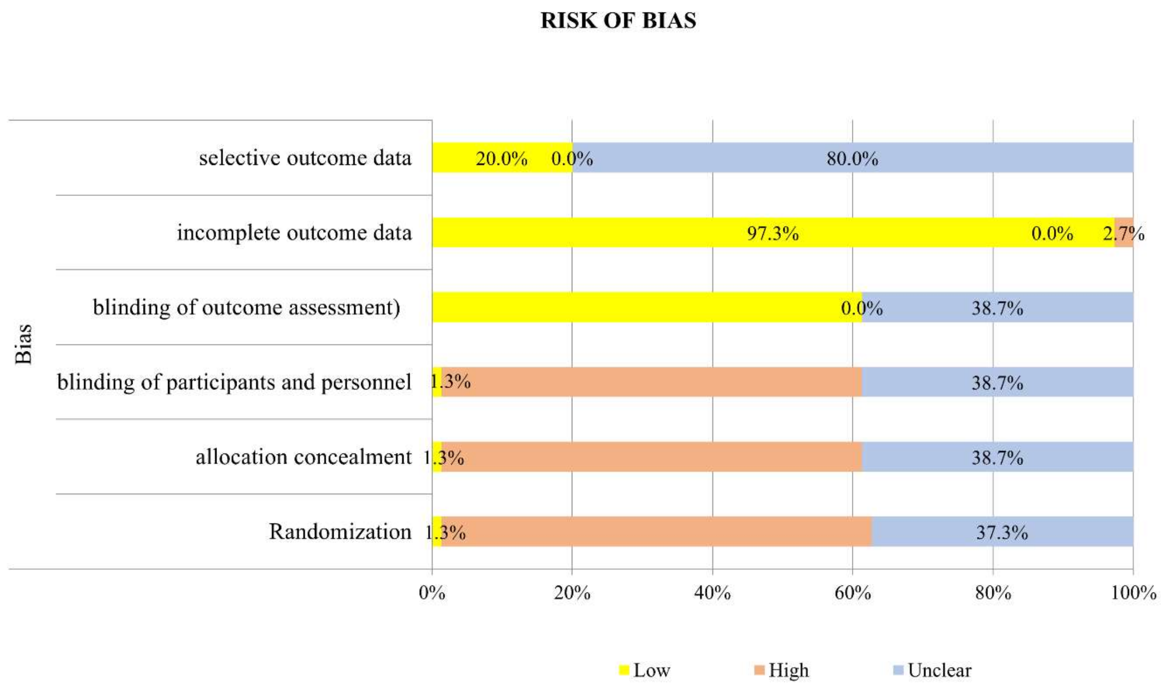

Supplementary Figure 2. Funnel plot to assess publication bias for all-grade neurotoxicity

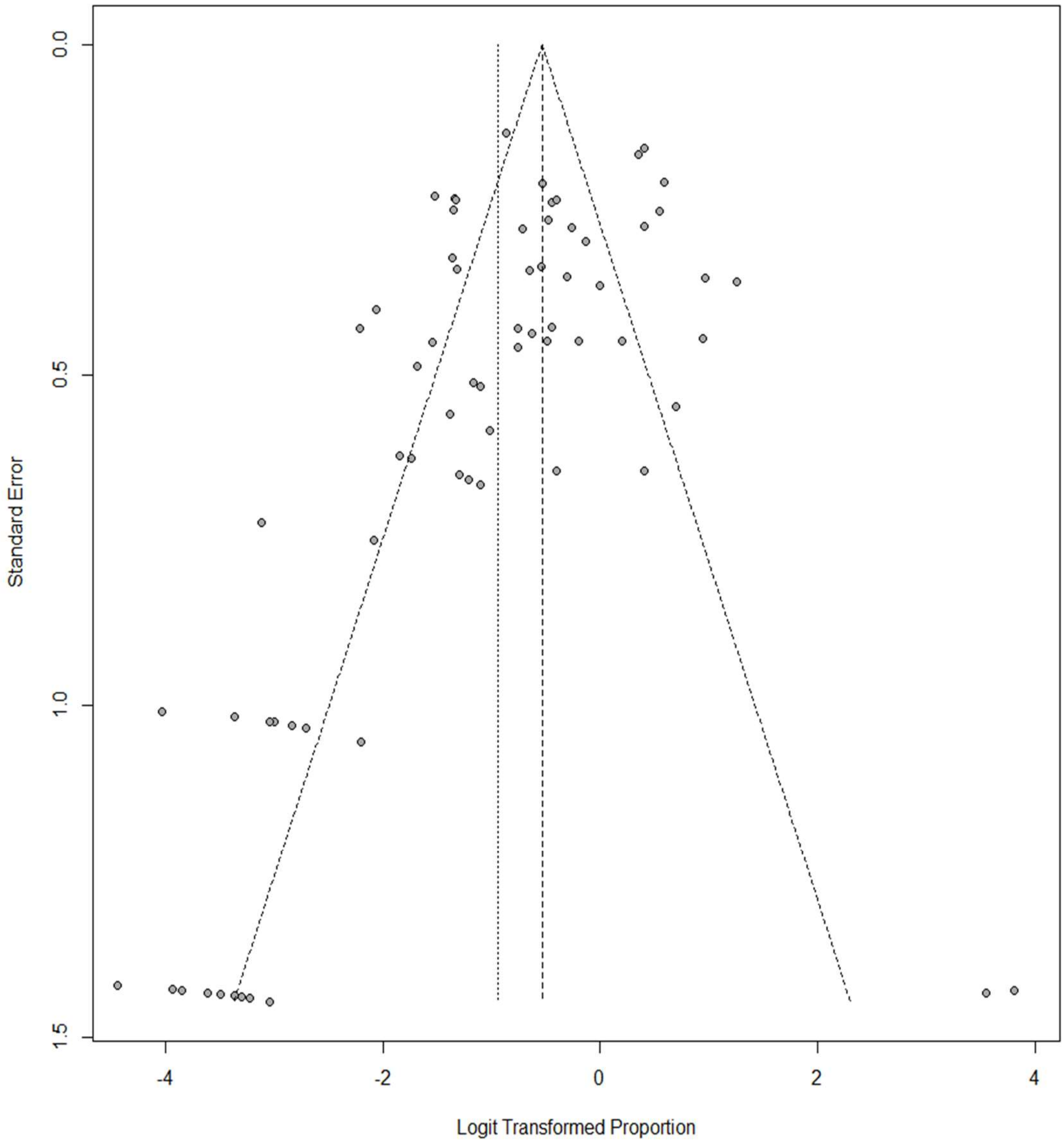

Supplementary Figure 3. Funnel plot to assess publication bias for high-grade neurotoxicity

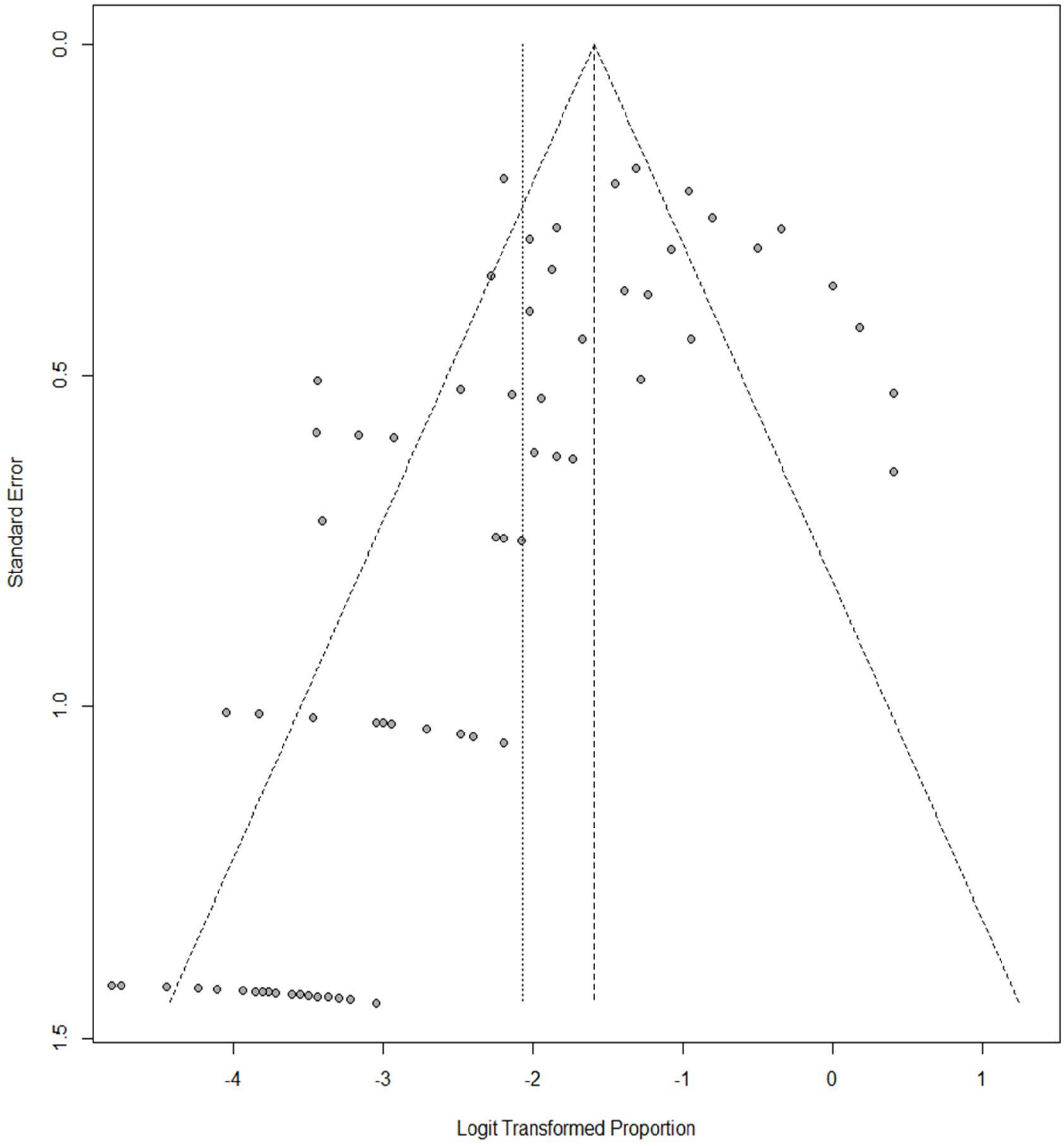

Supplementary Figure 4. Forest plots showing the pooled incidence of all-grade ICANS before (a) and after (b) removing outliers, high-grade ICANS before (c) and after (d) removing outliers. Studies in the boxes represent the identified outliers.

(a)

Author(s)	Event	Total	Proportion	95% CI
Abramson JS et al 2020 [1]	80	269	0.30	[0.24; 0.36]
An F et al 2020 [54]	2	47	0.04	[0.01; 0.15]
Baumeister SH et al 2019 [29]	0	12	0.00	[0.00; 0.26]
Benjamin R et al 2020 [30]	8	21	0.38	[0.18; 0.62]
Berdeja JG et al 2021 [31]	20	97	0.21	[0.13; 0.30]
Brudno JN et al 2018 [56]	1	16	0.06	[0.00; 0.30]
Brudno JN et al 2020 [55]	4	20	0.20	[0.06; 0.44]
Caimi PF et al 2021 [69]	5	20	0.25	[0.09; 0.49]
Cao J et al 2018 [32]	1	18	0.06	[0.00; 0.27]
Cohen AD et al 2019 [33]	8	25	0.32	[0.15; 0.54]
Cordoba S et al 2021 [70]	4	15	0.27	[0.08; 0.55]
Cornell RF et al 2021 [71]	3	14	0.21	[0.05; 0.51]
Curran KJ et al 2019 [34]	18	25	0.72	[0.51; 0.88]
Fan L et al 2022 [72]	0	10	0.00	[0.00; 0.31]
Fowler NH et al 2022 [82]	36	97	0.37	[0.28; 0.48]
Frey NV et al 2020 [57]	13	38	0.34	[0.20; 0.51]
Gardner RA et al 2017 [58]	21	45	0.47	[0.32; 0.62]
Geyer MB et al 2019 [35]	9	20	0.45	[0.23; 0.68]
Heng G et al 2020 [36]	4	10	0.40	[0.12; 0.74]
Jacobson CA et al 2022 [83]	87	148	0.59	[0.50; 0.67]
Jacoby E et al 2018 [37]	11	20	0.55	[0.32; 0.77]
Kato K et al 2022 [84]	0	16	0.00	[0.00; 0.21]
Kochenderfer JN et al 2017 [59]	22	22	1.00	[0.85; 1.00]
Lamure S et al 2021 [94]	23	60	0.38	[0.26; 0.52]
Li C et al 2021 [38]	1	30	0.03	[0.00; 0.17]
Liu H et al 2020 [60]	17	17	1.00	[0.80; 1.00]
Liu Y et al 2022 [93]	8	23	0.35	[0.16; 0.57]
Locke FL et al 2022 [92]	102	170	0.60	[0.52; 0.67]
Ma F et al 2019 [39]	6	10	0.60	[0.26; 0.88]
Magnani CF et al 2020 [40]	0	13	0.00	[0.00; 0.25]
Makita S et al 2022 [85]	1	10	0.10	[0.00; 0.45]
Maude SL et al 2018 [4]	30	75	0.40	[0.29; 0.52]
Mei H et al 2021 [73]	0	23	0.00	[0.00; 0.15]
Munshi NC et al 2021 [41]	23	128	0.18	[0.12; 0.26]
Myers RM et al 2021 [61]	29	74	0.39	[0.28; 0.51]
Neelapu SS et al 2017 [42]	65	101	0.64	[0.54; 0.74]
Neelapu SS et al 2022 [86]	29	40	0.72	[0.56; 0.85]
Pan J et al 2019 [43]	6	34	0.18	[0.07; 0.35]
Pan J et al 2021 [80]	3	20	0.15	[0.03; 0.38]
Park JH et al 2018 [14]	23	53	0.43	[0.30; 0.58]
Raje N et al 2019 [44]	14	33	0.42	[0.25; 0.61]
Ramos CA 2018 [62]	1	16	0.06	[0.00; 0.30]
Ramos CA et al 2020 [45]	0	42	0.00	[0.00; 0.08]
Roddie C et al 2021 [75]	4	20	0.20	[0.06; 0.44]
Sang W et al 2020 [15]	5	21	0.24	[0.08; 0.47]
Sauter CS et al 2019 [46]	10	15	0.67	[0.38; 0.88]
Schuster SJ et al 2019 [5]	23	111	0.21	[0.14; 0.29]
Shah BD et al 2021 [17]	33	55	0.60	[0.46; 0.73]
Shah BD et al 2021 [16]	35	45	0.78	[0.63; 0.89]
Shah NN et al 2020 [19]	7	22	0.32	[0.14; 0.55]
Shah NN et al 2020 [18]	19	58	0.33	[0.21; 0.46]
Shalabi H et al 2022 [76]	11	52	0.21	[0.11; 0.35]
Shi X et al 2022 [87]	0	10	0.00	[0.00; 0.31]
Siddiqi T et al 2022 [77]	9	23	0.39	[0.20; 0.61]
Singh H et al 2022 [63]	0	14	0.00	[0.00; 0.23]
Spiegel JY et al 2021 [64]	14	38	0.37	[0.22; 0.54]
Talleur A et al 2022 [78]	3	12	0.25	[0.05; 0.57]
Tong C et al 2020 [65]	6	61	0.10	[0.04; 0.20]
Turtle CJ et al 2016 [47]	15	30	0.50	[0.31; 0.69]
Wang D et al 2021 [20]	0	18	0.00	[0.00; 0.19]
Wang J et al 2022 [66]	2	18	0.11	[0.01; 0.35]
Wang M et al 2020 [91]	43	68	0.63	[0.51; 0.75]
Wang Y et al 2022 [67]	7	62	0.11	[0.05; 0.22]
Wei G et al 2021 [79]	0	16	0.00	[0.00; 0.21]
Wu J et al 2021 [90]	3	13	0.23	[0.05; 0.54]
Yan ZX et al 2019 [49]	1	10	0.10	[0.00; 0.45]
Ying Z et al 2019 [50]	0	25	0.00	[0.00; 0.14]
Ying Z et al 2021 [88]	12	59	0.20	[0.11; 0.33]
Ying Z et al 2022 [68]	1	22	0.05	[0.00; 0.23]
Zhang H et al 2022 [89]	3	22	0.14	[0.03; 0.35]
Zhang X et al 2020 [93]	23	110	0.21	[0.14; 0.30]
Zhang Y et al 2021 [51]	5	32	0.16	[0.05; 0.33]
Zhao WH et al 2018 [52]	1	57	0.02	[0.00; 0.09]
Zhou X et al 2020 [53]	1	21	0.05	[0.00; 0.24]
Total (common effect)		3137	0.37	[0.35; 0.39]
Total (random effect)			0.27	[0.22; 0.33]

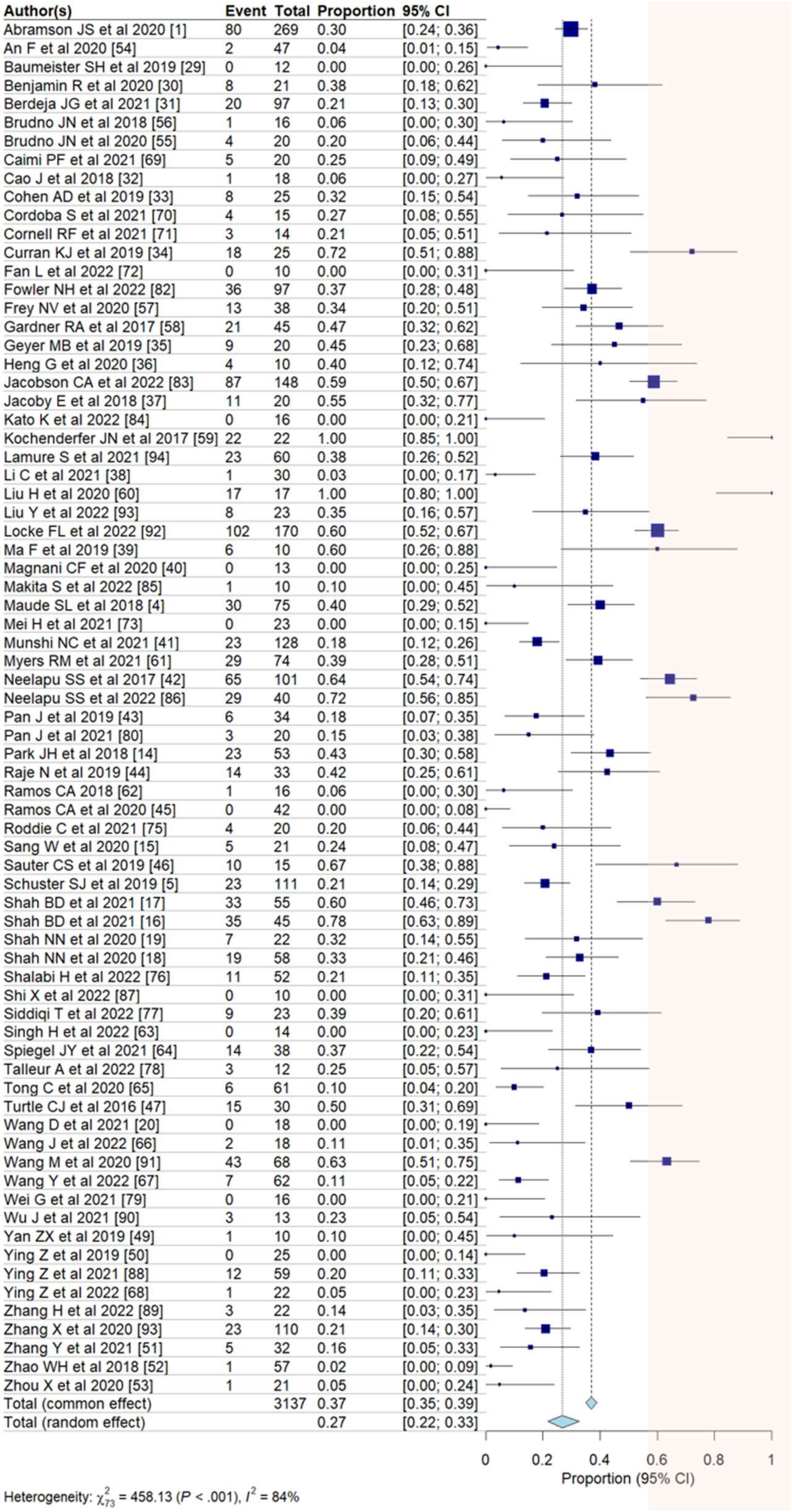

Heterogeneity: $\chi^2_{73} = 458.13$ (P < .001), I^2 = 84%

(b)

Author(s)	Event	Total	Proportion	95% CI
Abramson JS et al 2020 [1]	80	269	0.30	[0.24; 0.36]
An F et al 2020 [54]	2	47	0.04	[0.01; 0.15]
Baumeister SH et al 2019 [29]	0	12	0.00	[0.00; 0.26]
Benjamin R et al 2020 [30]	8	21	0.38	[0.18; 0.62]
Berdeja JG et al 2021 [31]	20	97	0.21	[0.13; 0.30]
Brudno JN et al 2018 [56]	1	16	0.06	[0.00; 0.30]
Brudno JN et al 2020 [55]	4	20	0.20	[0.06; 0.44]
Caimi PF et al 2021 [69]	5	20	0.25	[0.09; 0.49]
Cao J et al 2018 [32]	1	18	0.06	[0.00; 0.27]
Cohen AD et al 2019 [33]	8	25	0.32	[0.15; 0.54]
Cordoba S et al 2021 [70]	4	15	0.27	[0.08; 0.55]
Cornell RF et al 2021 [71]	3	14	0.21	[0.05; 0.51]
Fan L et al 2022 [72]	0	10	0.00	[0.00; 0.31]
Fowler NH et al 2022 [82]	36	97	0.37	[0.28; 0.48]
Frey NV et al 2020 [57]	13	38	0.34	[0.20; 0.51]
Gardner RA et al 2017 [58]	21	45	0.47	[0.32; 0.62]
Geyer MB et al 2019 [35]	9	20	0.45	[0.23; 0.68]
Heng G et al 2020 [36]	4	10	0.40	[0.12; 0.74]
Jacoby E et al 2018 [37]	11	20	0.55	[0.32; 0.77]
Kato K et al 2022 [84]	0	16	0.00	[0.00; 0.21]
Lamure S et al 2021 [94]	23	60	0.38	[0.26; 0.52]
Li C et al 2021 [38]	1	30	0.03	[0.00; 0.17]
Liu Y et al 2022 [93]	8	23	0.35	[0.16; 0.57]
Magnani CF et al 2020 [40]	0	13	0.00	[0.00; 0.25]
Makita S et al 2022 [85]	1	10	0.10	[0.00; 0.45]
Maude SL et al 2018 [4]	30	75	0.40	[0.29; 0.52]
Mei H et al 2021 [73]	0	23	0.00	[0.00; 0.15]
Munshi NC et al 2021 [41]	23	128	0.18	[0.12; 0.26]
Myers RM et al 2021 [61]	29	74	0.39	[0.28; 0.51]
Pan J et al 2019 [43]	6	34	0.18	[0.07; 0.35]
Pan J et al 2021 [80]	3	20	0.15	[0.03; 0.38]
Park JH et al 2018 [14]	23	53	0.43	[0.30; 0.58]
Raje N et al 2019 [44]	14	33	0.42	[0.25; 0.61]
Ramos CA 2018 [62]	1	16	0.06	[0.00; 0.30]
Ramos CA et al 2020 [45]	0	42	0.00	[0.00; 0.08]
Roddie C et al 2021 [75]	4	20	0.20	[0.06; 0.44]
Sang W et al 2020 [15]	5	21	0.24	[0.08; 0.47]
Schuster SJ et al 2019 [5]	23	111	0.21	[0.14; 0.29]
Shah NN et al 2020 [19]	7	22	0.32	[0.14; 0.55]
Shah NN et al 2020 [18]	19	58	0.33	[0.21; 0.46]
Shalabi H et al 2022 [76]	11	52	0.21	[0.11; 0.35]
Shi X et al 2022 [87]	0	10	0.00	[0.00; 0.31]
Siddiqi T et al 2022 [77]	9	23	0.39	[0.20; 0.61]
Singh H et al 2022 [63]	0	14	0.00	[0.00; 0.23]
Spiegel JY et al 2021 [64]	14	38	0.37	[0.22; 0.54]
Talleur A et al 2022 [78]	3	12	0.25	[0.05; 0.57]
Tong C et al 2020 [65]	6	61	0.10	[0.04; 0.20]
Turtle CJ et al 2016 [47]	15	30	0.50	[0.31; 0.69]
Wang D et al 2021 [20]	0	18	0.00	[0.00; 0.19]
Wang J et al 2022 [66]	2	18	0.11	[0.01; 0.35]
Wang Y et al 2022 [67]	7	62	0.11	[0.05; 0.22]
Wei G et al 2021 [79]	0	16	0.00	[0.00; 0.21]
Wu J et al 2021 [90]	3	13	0.23	[0.05; 0.54]
Yan ZX et al 2019 [49]	1	10	0.10	[0.00; 0.45]
Ying Z et al 2019 [50]	0	25	0.00	[0.00; 0.14]
Ying Z et al 2021 [88]	12	59	0.20	[0.11; 0.33]
Ying Z et al 2022 [68]	1	22	0.05	[0.00; 0.23]
Zhang H et al 2022 [89]	3	22	0.14	[0.03; 0.35]
Zhang X et al 2020 [93]	23	110	0.21	[0.14; 0.30]
Zhang Y et al 2021 [51]	5	32	0.16	[0.05; 0.33]
Zhao WH et al 2018 [52]	1	57	0.02	[0.00; 0.09]
Zhou X et al 2020 [53]	1	21	0.05	[0.00; 0.24]
Total (common effect)		2421	0.28	[0.26; 0.30]
Total (random effect)			0.22	[0.18; 0.26]

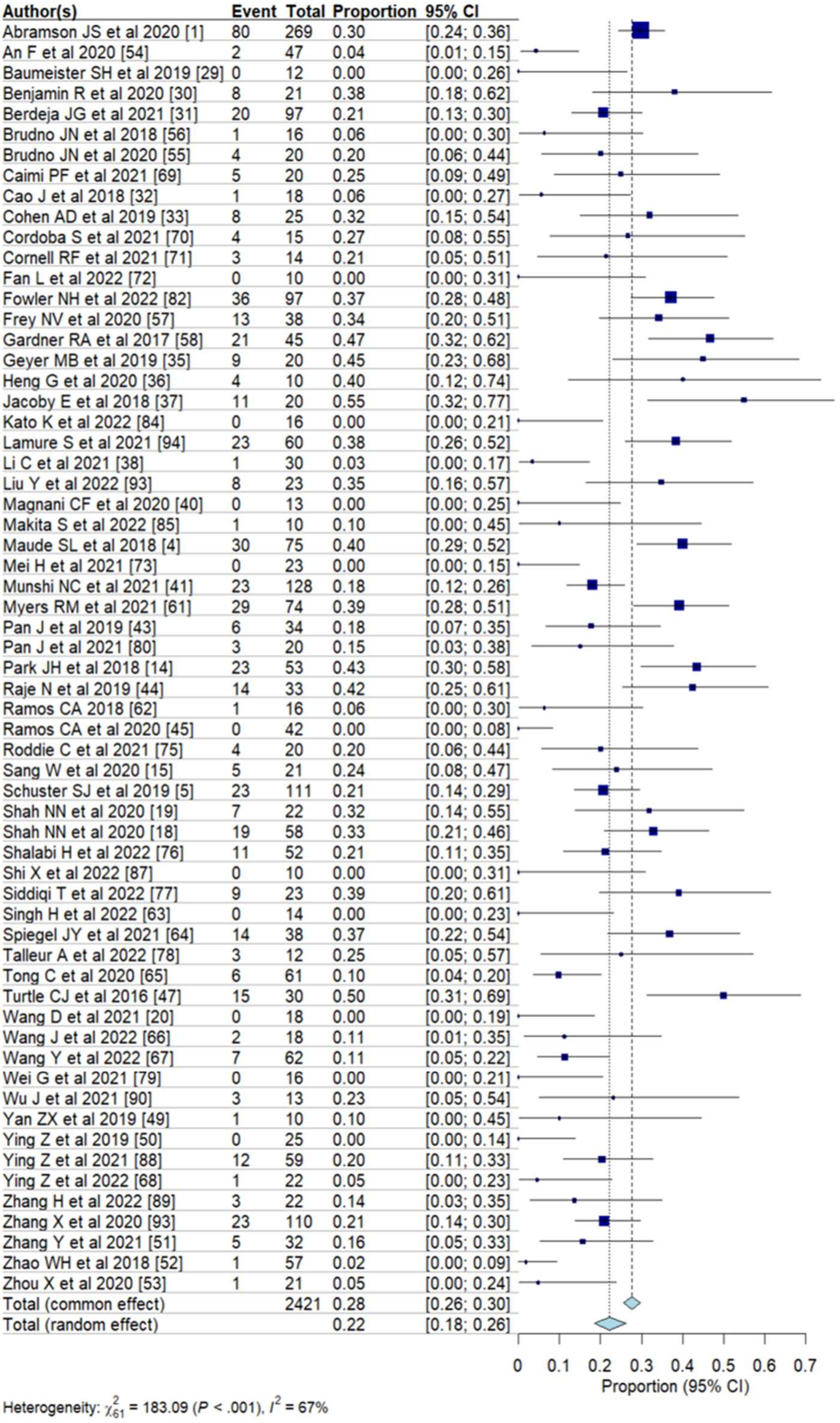

Heterogeneity: $\chi^2_{61} = 183.09$ ($P < .001$), $I^2 = 67\%$

(c)

Author(s)	Event	Total	Proportion	95% CI
Abramson JS et al 2020 [1]	27	269	0.10	[0.07; 0.14]
An F et al 2020 [54]	1	47	0.02	[0.00; 0.11]
Baumeister SH et al 2019 [29]	0	12	0.00	[0.00; 0.26]
Benjamin R et al 2020 [30]	0	21	0.00	[0.00; 0.16]
Berdeja JG et al 2021 [31]	9	97	0.09	[0.04; 0.17]
Brudno JN et al 2018 [56]	1	16	0.06	[0.00; 0.30]
Brudno JN et al 2020 [55]	1	20	0.05	[0.00; 0.25]
Caimi PF et al 2021 [69]	1	20	0.05	[0.00; 0.25]
Cohen AD et al 2019 [33]	3	25	0.12	[0.03; 0.31]
Cordoba S et al 2021 [70]	0	15	0.00	[0.00; 0.22]
Cornell RF et al 2021 [71]	0	14	0.00	[0.00; 0.23]
Curran KJ et al 2019 [34]	7	25	0.28	[0.12; 0.49]
Fan L et al 2022 [72]	0	10	0.00	[0.00; 0.31]
Fowler NH et al 2022 [82]	3	97	0.03	[0.01; 0.09]
Frey NV et al 2020 [57]	6	38	0.16	[0.06; 0.31]
Gardner RA et al 2017 [58]	9	45	0.20	[0.10; 0.35]
Geyer MB et al 2019 [35]	2	20	0.10	[0.01; 0.32]
Jacobson CA et al 2022 [83]	28	148	0.19	[0.13; 0.26]
Kato K et al 2022 [84]	0	16	0.00	[0.00; 0.21]
Kochenderfer JN et al 2017 [59]	12	22	0.55	[0.32; 0.76]
Lamure S et al 2021 [94]	7	60	0.12	[0.05; 0.23]
Li C et al 2021 [38]	0	30	0.00	[0.00; 0.12]
Liu H et al 2020 [60]	0	17	0.00	[0.00; 0.20]
Liu Y et al 2022 [93]	5	23	0.22	[0.07; 0.44]
Locke FL et al 2022 [92]	36	170	0.21	[0.15; 0.28]
Ma F et al 2019 [39]	6	10	0.60	[0.26; 0.88]
Magnani CF et al 2020 [40]	0	13	0.00	[0.00; 0.25]
Makita S et al 2022 [85]	0	10	0.00	[0.00; 0.31]
Maude SL et al 2018 [4]	10	75	0.13	[0.07; 0.23]
Mei H et al 2021 [73]	0	23	0.00	[0.00; 0.15]
Munshi NC et al 2021 [41]	4	128	0.03	[0.01; 0.08]
Myers RM et al 2021 [61]	3	74	0.04	[0.01; 0.11]
Neelapu SS et al 2017 [42]	28	101	0.28	[0.19; 0.38]
Neelapu SS et al 2022 [86]	9	40	0.22	[0.11; 0.38]
Pan J et al 2019 [43]	0	34	0.00	[0.00; 0.10]
Pan J et al 2021 [80]	0	20	0.00	[0.00; 0.17]
Park JH et al 2018 [14]	22	53	0.42	[0.28; 0.56]
Raje N et al 2019 [44]	1	33	0.03	[0.00; 0.16]
Ramos CA 2018 [62]	1	16	0.06	[0.00; 0.30]
Ramos CA et al 2020 [45]	0	42	0.00	[0.00; 0.08]
Roddie C et al 2021 [75]	3	20	0.15	[0.03; 0.38]
Sang W et al 2020 [15]	2	21	0.10	[0.01; 0.30]
Sauter CS et al 2019 [46]	9	15	0.60	[0.32; 0.84]
Schuster SJ et al 2019 [5]	13	111	0.12	[0.06; 0.19]
Shah BD et al 2021 [17]	14	55	0.25	[0.15; 0.39]
Shah BD et al 2021 [16]	17	45	0.38	[0.24; 0.53]
Shah NN et al 2020 [19]	3	22	0.14	[0.03; 0.35]
Shah NN et al 2020 [18]	1	58	0.02	[0.00; 0.09]
Shalabi H et al 2022 [76]	4	52	0.08	[0.02; 0.19]
Shi X et al 2022 [87]	0	10	0.00	[0.00; 0.31]
Siddiqi T et al 2022 [77]	5	23	0.22	[0.07; 0.44]
Singh H et al 2022 [63]	0	14	0.00	[0.00; 0.23]
Spiegel JY et al 2021 [64]	4	38	0.11	[0.03; 0.25]
Talleur A et al 2022 [78]	1	12	0.08	[0.00; 0.38]
Tong C et al 2020 [65]	0	61	0.00	[0.00; 0.06]
Turtle CJ et al 2016 [47]	15	30	0.50	[0.31; 0.69]
Wang D et al 2021 [20]	0	18	0.00	[0.00; 0.19]
Wang J et al 2022 [66]	2	18	0.11	[0.01; 0.35]
Wang M et al 2020 [91]	21	68	0.31	[0.20; 0.43]
Wang Y et al 2022 [67]	2	62	0.03	[0.00; 0.11]
Wei G et al 2021 [79]	0	16	0.00	[0.00; 0.21]
Wu J et al 2021 [90]	1	13	0.08	[0.00; 0.36]
Yan ZX et al 2019 [49]	1	10	0.10	[0.00; 0.45]
Ying Z et al 2019 [50]	0	25	0.00	[0.00; 0.14]
Ying Z et al 2021 [88]	3	59	0.05	[0.01; 0.14]
Ying Z et al 2022 [68]	1	22	0.05	[0.00; 0.23]
Zhang H et al 2022 [89]	0	22	0.00	[0.00; 0.15]
Zhang X et al 2020 [93]	15	110	0.14	[0.08; 0.21]
Zhang Y et al 2021 [51]	4	32	0.12	[0.04; 0.29]
Zhao WH et al 2018 [52]	0	57	0.00	[0.00; 0.06]
Zhou X et al 2020 [53]	1	21	0.05	[0.00; 0.24]
Total (common effect)		3089	0.17	[0.16; 0.19]
Total (random effect)			0.11	[0.08; 0.14]

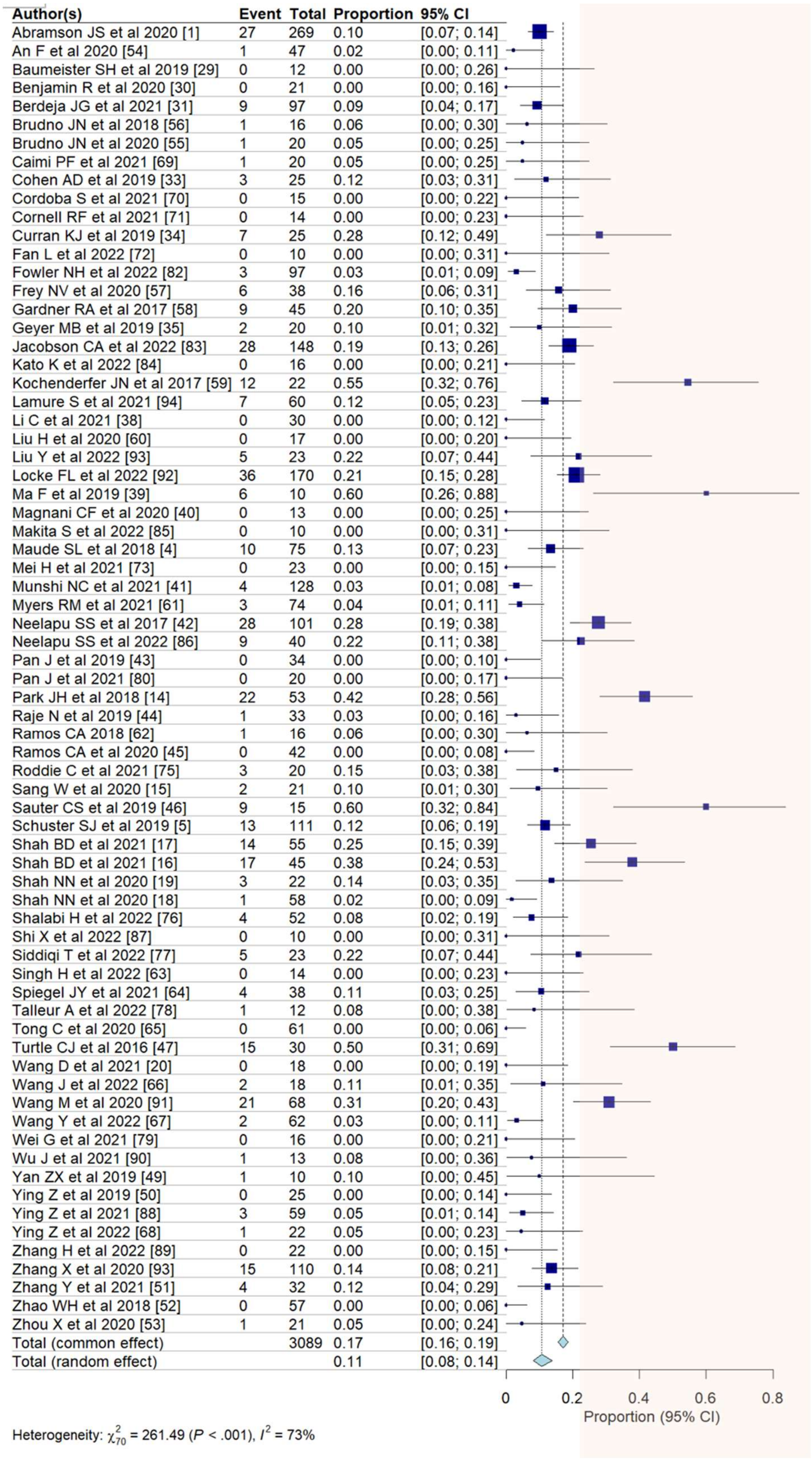

Heterogeneity: $\chi^2_{70} = 261.49$ ($P < .001$), $I^2 = 73\%$

(d)

Author(s)	Event	Total	Proportion	95% CI
Abramson JS et al 2020 [1]	27	269	0.10	[0.07; 0.14]
An F et al 2020 [54]	1	47	0.02	[0.00; 0.11]
Baumeister SH et al 2019 [29]	0	12	0.00	[0.00; 0.26]
Benjamin R et al 2020 [30]	0	21	0.00	[0.00; 0.16]
Berdeja JG et al 2021 [31]	9	97	0.09	[0.04; 0.17]
Brudno JN et al 2018 [56]	1	16	0.06	[0.00; 0.30]
Brudno JN et al 2020 [55]	1	20	0.05	[0.00; 0.25]
Caimi PF et al 2021 [69]	1	20	0.05	[0.00; 0.25]
Cohen AD et al 2019 [33]	3	25	0.12	[0.03; 0.31]
Cordoba S et al 2021 [70]	0	15	0.00	[0.00; 0.22]
Cornell RF et al 2021 [71]	0	14	0.00	[0.00; 0.23]
Fan L et al 2022 [72]	0	10	0.00	[0.00; 0.31]
Fowler NH et al 2022 [82]	3	97	0.03	[0.01; 0.09]
Frey NV et al 2020 [57]	6	38	0.16	[0.06; 0.31]
Gardner RA et al 2017 [58]	9	45	0.20	[0.10; 0.35]
Geyer MB et al 2019 [35]	2	20	0.10	[0.01; 0.32]
Jacobson CA et al 2022 [83]	28	148	0.19	[0.13; 0.26]
Kato K et al 2022 [84]	0	16	0.00	[0.00; 0.21]
Lamure S et al 2021 [94]	7	60	0.12	[0.05; 0.23]
Li C et al 2021 [38]	0	30	0.00	[0.00; 0.12]
Liu H et al 2020 [60]	0	17	0.00	[0.00; 0.20]
Liu Y et al 2022 [93]	5	23	0.22	[0.07; 0.44]
Locke FL et al 2022 [92]	36	170	0.21	[0.15; 0.28]
Magnani CF et al 2020 [40]	0	13	0.00	[0.00; 0.25]
Makita S et al 2022 [85]	0	10	0.00	[0.00; 0.31]
Maude SL et al 2018 [4]	10	75	0.13	[0.07; 0.23]
Mei H et al 2021 [73]	0	23	0.00	[0.00; 0.15]
Munshi NC et al 2021 [41]	4	128	0.03	[0.01; 0.08]
Myers RM et al 2021 [61]	3	74	0.04	[0.01; 0.11]
Pan J et al 2019 [43]	0	34	0.00	[0.00; 0.10]
Pan J et al 2021 [80]	0	20	0.00	[0.00; 0.17]
Raje N et al 2019 [44]	1	33	0.03	[0.00; 0.16]
Ramos CA 2018 [62]	1	16	0.06	[0.00; 0.30]
Ramos CA et al 2020 [45]	0	42	0.00	[0.00; 0.08]
Roddie C et al 2021 [75]	3	20	0.15	[0.03; 0.38]
Sang W et al 2020 [15]	2	21	0.10	[0.01; 0.30]
Schuster SJ et al 2019 [5]	13	111	0.12	[0.06; 0.19]
Shah NN et al 2020 [19]	3	22	0.14	[0.03; 0.35]
Shah NN et al 2020 [18]	1	58	0.02	[0.00; 0.09]
Shalabi H et al 2022 [76]	4	52	0.08	[0.02; 0.19]
Shi X et al 2022 [87]	0	10	0.00	[0.00; 0.31]
Siddiqi T et al 2022 [77]	5	23	0.22	[0.07; 0.44]
Singh H et al 2022 [63]	0	14	0.00	[0.00; 0.23]
Spiegel JY et al 2021 [64]	4	38	0.11	[0.03; 0.25]
Talleur A et al 2022 [78]	1	12	0.08	[0.00; 0.38]
Tong C et al 2020 [65]	0	61	0.00	[0.00; 0.06]
Wang D et al 2021 [20]	0	18	0.00	[0.00; 0.19]
Wang J et al 2022 [66]	2	18	0.11	[0.01; 0.35]
Wang Y et al 2022 [67]	2	62	0.03	[0.00; 0.11]
Wei G et al 2021 [79]	0	16	0.00	[0.00; 0.21]
Wu J et al 2021 [90]	1	13	0.08	[0.00; 0.36]
Yan ZX et al 2019 [49]	1	10	0.10	[0.00; 0.45]
Ying Z et al 2019 [50]	0	25	0.00	[0.00; 0.14]
Ying Z et al 2021 [88]	3	59	0.05	[0.01; 0.14]
Ying Z et al 2022 [68]	1	22	0.05	[0.00; 0.23]
Zhang H et al 2022 [89]	0	22	0.00	[0.00; 0.15]
Zhang X et al 2020 [93]	15	110	0.14	[0.08; 0.21]
Zhang Y et al 2021 [51]	4	32	0.12	[0.04; 0.29]
Zhao WH et al 2018 [52]	0	57	0.00	[0.00; 0.06]
Zhou X et al 2020 [53]	1	21	0.05	[0.00; 0.24]
Total (common effect)		2625	0.11	[0.10; 0.13]
Total (random effect)			0.08	[0.07; 0.10]

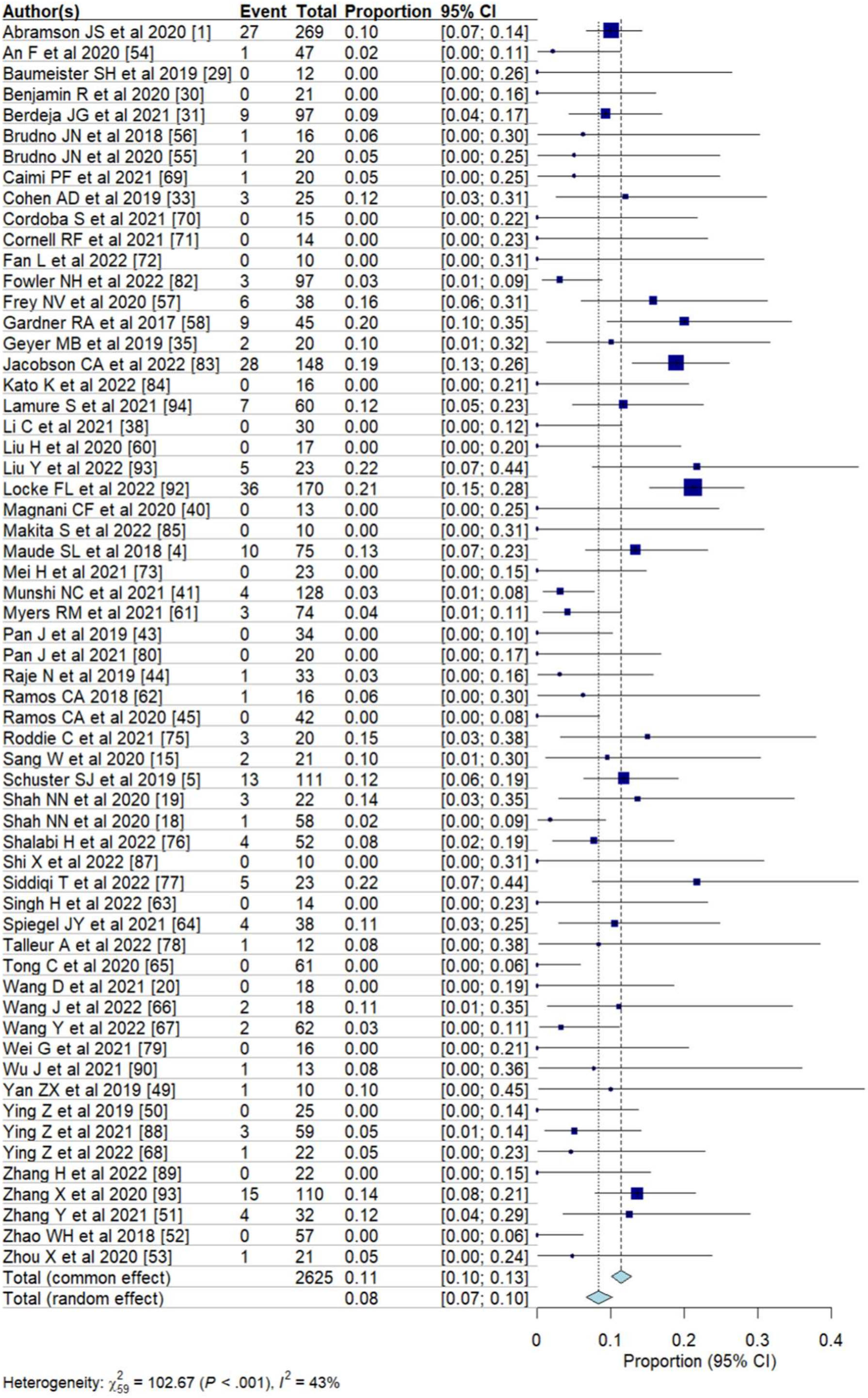

Heterogeneity: $\chi^2_{59} = 102.67$ ($P < .001$), $I^2 = 43\%$

Supplementary Figure 5. Forest plots showing the pooled incidence of all-grade ICANS (a) and high-grade (b) in real-world data (76-87).

(a)

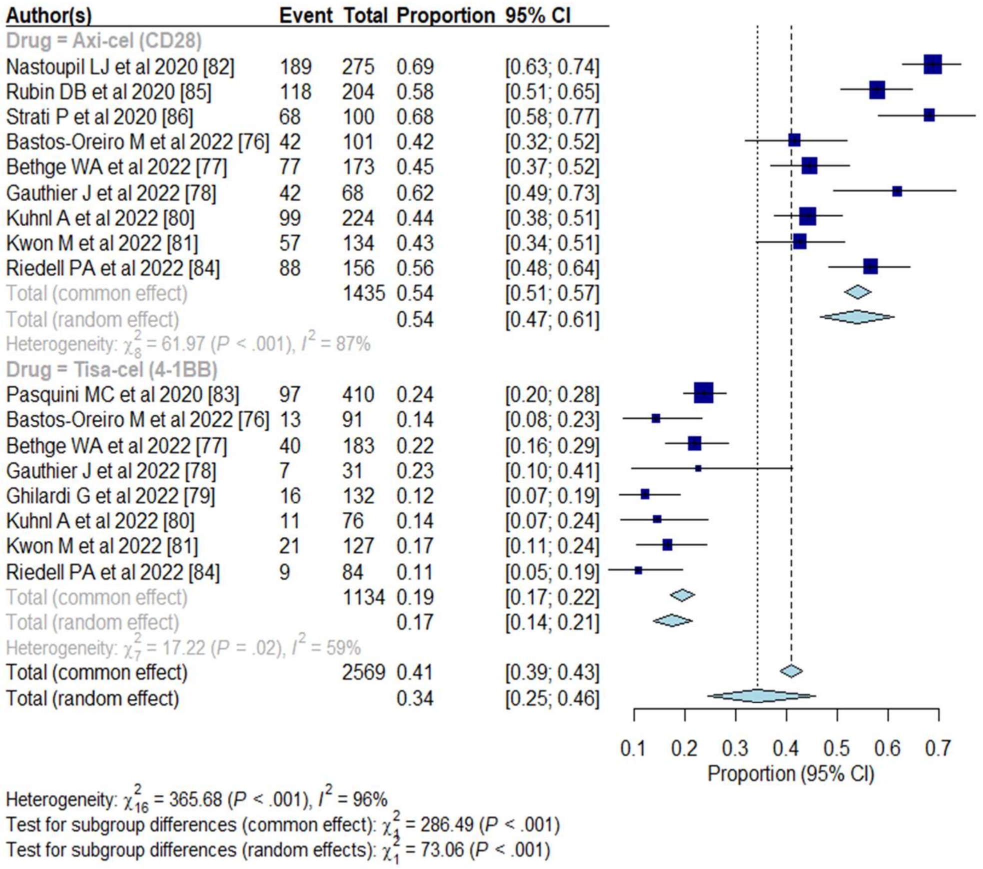

(b)

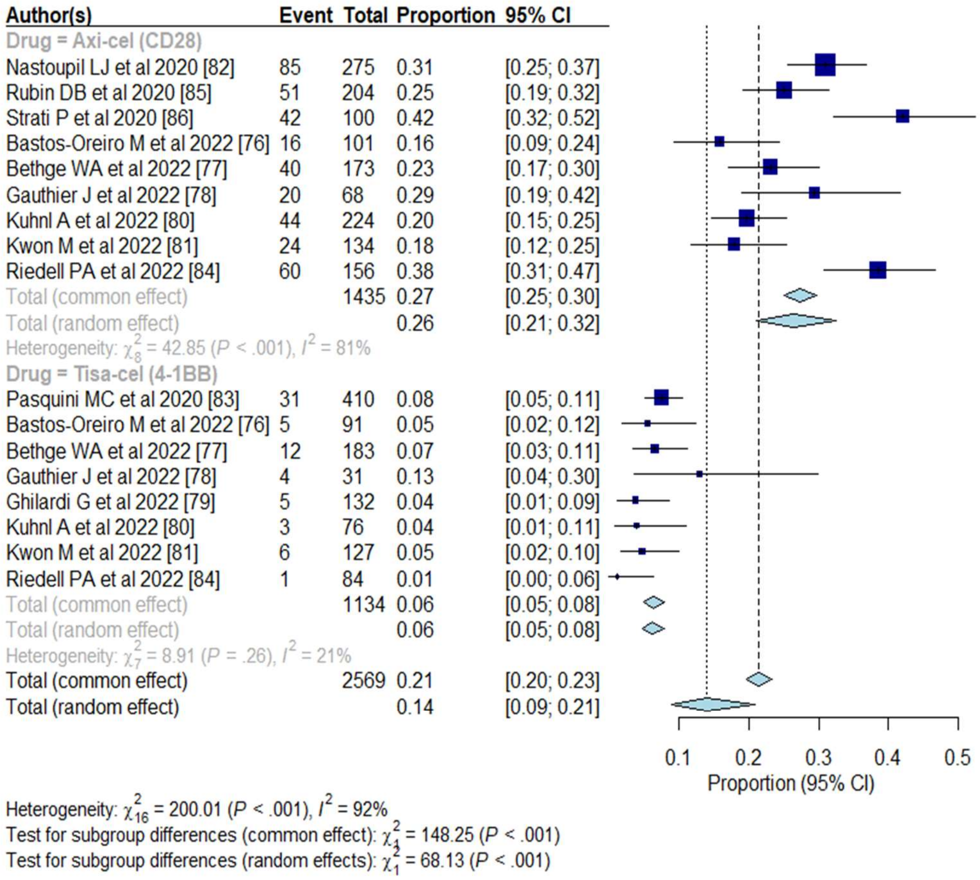
Author(s) Event Total Proportion 95% CI
Drug = Axi-cel (CD28)
Nastoupil LJ et al 2020 [82] 85 275 0.31 [0.25; 0.37]
Rubin DB et al 2020 [85] 51 204 0.25 [0.19; 0.32]
Strati P et al 2020 [86] 42 100 0.42 [0.32; 0.52]
Bastos-Oreiro M et al 2022 [76] 16 101 0.16 [0.09; 0.24]
Bethge WA et al 2022 [77] 40 173 0.23 [0.17; 0.30]
Gauthier J et al 2022 [78] 20 68 0.29 [0.19; 0.42]
Kuhnl A et al 2022 [80] 44 224 0.20 [0.15; 0.25]
Kwon M et al 2022 [81] 24 134 0.18 [0.12; 0.25]
Riedell PA et al 2022 [84] 60 156 0.38 [0.31; 0.47]
Total (common effect) 1435 0.27 [0.25; 0.30]
Total (random effect) 0.26 [0.21; 0.32]
Heterogeneity: χ^2_8 = 42.85 (P < .001), I^2 = 81%
Drug = Tisa-cel (4-1BB)
Pasquini MC et al 2020 [83] 31 410 0.08 [0.05; 0.11]
Bastos-Oreiro M et al 2022 [76] 5 91 0.05 [0.02; 0.12]
Bethge WA et al 2022 [77] 12 183 0.07 [0.03; 0.11]
Gauthier J et al 2022 [78] 4 31 0.13 [0.04; 0.30]
Ghilardi G et al 2022 [79] 5 132 0.04 [0.01; 0.09]
Kuhnl A et al 2022 [80] 3 76 0.04 [0.01; 0.11]
Kwon M et al 2022 [81] 6 127 0.05 [0.02; 0.10]
Riedell PA et al 2022 [84] 1 84 0.01 [0.00; 0.06]
Total (common effect) 1134 0.06 [0.05; 0.08]
Total (random effect) 0.06 [0.05; 0.08]
Heterogeneity: χ^2_7 = 8.91 (P = .26), I^2 = 21%
Total (common effect) 2569 0.21 [0.20; 0.23]
Total (random effect) 0.14 [0.09; 0.21]
0.1 0.2 0.3 0.4 0.5
Proportion (95% CI)
Heterogeneity: χ^2_{16} = 200.01 (P < .001), I^2 = 92%
Test for subgroup differences (common effect): χ^2_1 = 148.25 (P < .001)
Test for subgroup differences (random effects): χ^2_1 = 68.13 (P < .001)

Reference)

1. Abramson JS, Palomba ML, Gordon LI, Lunning MA, Wang M, Arnason J, et al. Lisocabtagene maraleucel for patients with relapsed or refractory large B-cell lymphomas (TRANSCEND NHL 001): a multicentre seamless design study. *Lancet*. (2020) 396:839-52. doi: 10.1016/s0140-6736(20)31366-0

2. An F, Wang H, Liu Z, Wu F, Zhang J, Tao Q, et al. Influence of patient characteristics on chimeric antigen receptor T cell therapy in B-cell acute lymphoblastic leukemia. *Nature communications*. (2020) 11:5928. doi: 10.1038/s41467-020-19774-x

3. Baumeister SH, Murad J, Werner L, Daley H, Trebeden-Negre H, Gicobi JK, et al. Phase I Trial of Autologous CAR T Cells Targeting NKG2D Ligands in Patients with AML/MDS and Multiple Myeloma. *Cancer Immunol Res*. (2019) 7:100-12. doi: 10.1158/2326-6066.Cir-18-0307

4. Benjamin R, Graham C, Yallop D, Jozwik A, Mirci-Danicar OC, Lucchini G, et al. Genome-edited, donor-derived allogeneic anti-CD19 chimeric antigen receptor T cells in paediatric and adult B-cell acute lymphoblastic leukaemia: results of two phase 1 studies. *Lancet*. (2020) 396:1885-94. doi: 10.1016/s0140-6736(20)32334-5

5. Berdeja JG, Madduri D, Usmani SZ, Jakubowiak A, Agha M, Cohen AD, et al. Ciltacabtagene autoleucel, a B-cell maturation antigen-directed chimeric antigen receptor T-cell therapy in patients with relapsed or refractory multiple myeloma (CARTITUDE-1): a phase 1b/2 open-label study. *Lancet*. (2021). doi: 10.1016/s0140-6736(21)00933-8. doi: 10.1016/s0140-6736(21)00933-8

6. Brudno JN, Maric I, Hartman SD, Rose JJ, Wang M, Lam N, et al. T Cells Genetically Modified to Express an Anti-B-Cell Maturation Antigen Chimeric Antigen Receptor Cause Remissions of Poor-Prognosis Relapsed Multiple Myeloma. *Journal of clinical oncology : official journal of the American Society of Clinical Oncology*. (2018) 36:2267-80. doi: 10.1200/jco.2018.77.8084

7. Brudno JN, Lam N, Vanasse D, Shen YW, Rose JJ, Rossi J, et al. Safety and feasibility of

anti-CD19 CAR T cells with fully human binding domains in patients with B-cell lymphoma. *Nature medicine*. (2020) 26:270-80. doi: 10.1038/s41591-019-0737-3

8. Caimi PF, Pacheco Sanchez G, Sharma A, Otegbeye F, Ahmed N, Rojas P, et al. Prophylactic Tocilizumab Prior to Anti-CD19 CAR-T Cell Therapy for Non-Hodgkin Lymphoma. *Frontiers in immunology*. (2021) 12:745320. doi: 10.3389/fimmu.2021.745320

9. Cao J, Wang G, Cheng H, Wei C, Qi K, Sang W, et al. Potent anti-leukemia activities of humanized CD19-targeted Chimeric antigen receptor T (CAR-T) cells in patients with relapsed/refractory acute lymphoblastic leukemia. *Am J Hematol*. (2018) 93:851-8. doi: 10.1002/ajh.25108

10. Cohen AD, Garfall AL, Stadtmauer EA, Melenhorst JJ, Lacey SF, Lancaster E, et al. B cell maturation antigen-specific CAR T cells are clinically active in multiple myeloma. *J Clin Invest*. (2019) 129:2210-21. doi: 10.1172/jci126397

11. Cordoba S, Onuoha S, Thomas S, Pignataro DS, Hough R, Ghorashian S, et al. CAR T cells with dual targeting of CD19 and CD22 in pediatric and young adult patients with relapsed or refractory B cell acute lymphoblastic leukemia: a phase 1 trial. *Nature medicine*. (2021) 27:1797-805. doi: 10.1038/s41591-021-01497-1

12. Cornell RF, Bishop MR, Kumar S, Giralt SA, Nooka AK, Larson SM, et al. A phase 1, multicenter study evaluating the safety and efficacy of KITE-585, an autologous anti-BCMA CAR T-cell therapy, in patients with relapsed/refractory multiple myeloma. *American journal of cancer research*. (2021) 11:3285-93

13. Curran KJ, Margossian SP, Kernan NA, Silverman LB, Williams DA, Shukla N, et al. Toxicity and response after CD19-specific CAR T-cell therapy in pediatric/young adult relapsed/refractory B-ALL. *Blood*. (2019) 134:2361-8. doi: 10.1182/blood.2019001641

14. Fan L, Wang L, Cao L, Zhu H, Xu W, Li J. Phase I study of CBM.CD19 chimeric antigen receptor T cell in the treatment of refractory diffuse large B-cell lymphoma in Chinese patients. *Frontiers of medicine*. (2022) 16:285-94. doi: 10.1007/s11684-021-0843-8

15. Fowler NH, Dickinson M, Dreyling M, Martinez-Lopez J, Kolstad A, Butler J, et al.

Tisagenlecleucel in adult relapsed or refractory follicular lymphoma: the phase 2 ELARA trial. *Nature medicine*. (2022) 28:325-32. doi: 10.1038/s41591-021-01622-0

16. Frey NV, Gill S, Hexner EO, Schuster S, Nasta S, Loren A, et al. Long-Term Outcomes From a Randomized Dose Optimization Study of Chimeric Antigen Receptor Modified T Cells in Relapsed Chronic Lymphocytic Leukemia. *Journal of clinical oncology : official journal of the American Society of Clinical Oncology*. (2020) 38:2862-71. doi: 10.1200/jco.19.03237

17. Gardner RA, Finney O, Annesley C, Brakke H, Summers C, Leger K, et al. Intent-to-treat leukemia remission by CD19 CAR T cells of defined formulation and dose in children and young adults. *Blood*. (2017) 129:3322-31. doi: 10.1182/blood-2017-02-769208

18. Geyer MB, Rivière I, Sénéchal B, Wang X, Wang Y, Purdon TJ, et al. Safety and tolerability of conditioning chemotherapy followed by CD19-targeted CAR T cells for relapsed/refractory CLL. *JCI Insight*. (2019) 5. doi: 10.1172/jci.insight.122627

19. Heng G, Jia J, Li S, Fu G, Wang M, Qin D, et al. Sustained Therapeutic Efficacy of Humanized Anti-CD19 Chimeric Antigen Receptor T Cells in Relapsed/Refractory Acute Lymphoblastic Leukemia. *Clin Cancer Res*. (2020) 26:1606-15. doi: 10.1158/1078-0432.Ccr-19-1339

20. Jacobson CA, Chavez JC, Sehgal AR, William BM, Munoz J, Salles G, et al. Axicabtagene ciloleucel in relapsed or refractory indolent non-Hodgkin lymphoma (ZUMA-5): a single-arm, multicentre, phase 2 trial. *The Lancet Oncology*. (2022) 23:91-103. doi: 10.1016/s1470-2045(21)00591-x

21. Jacoby E, Bielorai B, Avigdor A, Itzhaki O, Hutt D, Nussboim V, et al. Locally produced CD19 CAR T cells leading to clinical remissions in medullary and extramedullary relapsed acute lymphoblastic leukemia. *Am J Hematol*. (2018) 93:1485-92. doi: 10.1002/ajh.25274

22. Kato K, Makita S, Goto H, Kanda J, Fujii N, Shimada K, et al. Phase 2 study of axicabtagene ciloleucel in Japanese patients with relapsed or refractory large B-cell lymphoma. *International journal of clinical oncology*. (2022) 27:213-23. doi: 10.1007/s10147-021-02033-4

23. Kochenderfer JN, Somerville RPT, Lu T, Shi V, Bot A, Rossi J, et al. Lymphoma Remissions Caused by Anti-CD19 Chimeric Antigen Receptor T Cells Are Associated With High Serum Interleukin-15 Levels. *Journal of clinical oncology : official journal of the American Society of Clinical Oncology*. (2017) 35:1803-13. doi: 10.1200/jco.2016.71.3024

24. Lamure S, Van Laethem F, De Verbizier D, Lozano C, Gehlkopf E, Tudesq JJ, et al. Clinical and Product Features Associated with Outcome of DLBCL Patients to CD19-Targeted CAR T-Cell Therapy. *Cancers*. (2021) 13. doi: 10.3390/cancers13174279

25. Ortíz-Maldonado V, Rives S, Castellà M, Alonso-Saladrigues A, Benítez-Ribas D, Caballero-Baños M, et al. CART19-BE-01: A Multicenter Trial of ARI-0001 Cell Therapy in Patients with CD19(+) Relapsed/Refractory Malignancies. *Molecular therapy : the journal of the American Society of Gene Therapy*. (2021) 29:636-44. doi: 10.1016/j.ymthe.2020.09.027

26. Liu H, Lei W, Zhang C, Yang C, Wei J, Guo Q, et al. CD19-specific CAR T Cells that Express a PD-1/CD28 Chimeric Switch-Receptor are Effective in Patients with PD-L1-positive B-Cell Lymphoma. *Clin Cancer Res*. (2021) 27:473-84. doi: 10.1158/1078-0432.Ccr-20-1457

27. Liu Y, Deng B, Hu B, Zhang W, Zhu Q, Liu Y, et al. Sequential different B-cell antigen-targeted CAR T-cell therapy for pediatric refractory/relapsed Burkitt lymphoma. *Blood Adv*. (2022) 6:717-30. doi: 10.1182/bloodadvances.2021004557

28. Locke FL, Miklos DB, Jacobson CA, Perales MA, Kersten MJ, Oluwole OO, et al. Axicabtagene Ciloleucel as Second-Line Therapy for Large B-Cell Lymphoma. *The New England journal of medicine*. (2022) 386:640-54. doi: 10.1056/NEJMoa2116133

29. Ma F, Ho JY, Du H, Xuan F, Wu X, Wang Q, et al. Evidence of long-lasting anti-CD19 activity of engrafted CD19 chimeric antigen receptor-modified T cells in a phase I study targeting pediatrics with acute lymphoblastic leukemia. *Hematol Oncol*. (2019) 37:601-8. doi: 10.1002/hon.2672

30. Magnani CF, Gaipa G, Lussana F, Belotti D, Gritti G, Napolitano S, et al. Sleeping Beauty-engineered CAR T cells achieve antileukemic activity without severe toxicities. *J Clin Invest*.

(2020) 130:6021-33. doi: 10.1172/jci138473

31. Makita S, Yamamoto G, Maruyama D, Asano-Mori Y, Kaji D, Ananthakrishnan R, et al. Phase 2 results of lisocabtagene maraleucel in Japanese patients with relapsed/refractory aggressive B-cell non-Hodgkin lymphoma. *Cancer medicine*. (2022). doi: 10.1002/cam4.4820. doi: 10.1002/cam4.4820

32. Maude SL, Laetsch TW, Buechner J, Rives S, Boyer M, Bittencourt H, et al. Tisagenlecleucel in Children and Young Adults with B-Cell Lymphoblastic Leukemia. *The New England journal of medicine*. (2018) 378:439-48. doi: 10.1056/NEJMoa1709866

33. Mei H, Li C, Jiang H, Zhao X, Huang Z, Jin D, et al. A bispecific CAR-T cell therapy targeting BCMA and CD38 in relapsed or refractory multiple myeloma. *Journal of hematology & oncology*. (2021) 14:161. doi: 10.1186/s13045-021-01170-7

34. Munshi NC, Anderson LD, Jr., Shah N, Madduri D, Berdeja J, Lonial S, et al. Idecabtagene Vicleucel in Relapsed and Refractory Multiple Myeloma. *The New England journal of medicine*. (2021) 384:705-16. doi: 10.1056/NEJMoa2024850

35. Myers RM, Li Y, Barz Leahy A, Barrett DM, Teachey DT, Callahan C, et al. Humanized CD19-Targeted Chimeric Antigen Receptor (CAR) T Cells in CAR-Naive and CAR-Exposed Children and Young Adults With Relapsed or Refractory Acute Lymphoblastic Leukemia. *Journal of clinical oncology : official journal of the American Society of Clinical Oncology*. (2021) 39:3044-55. doi: 10.1200/jco.20.03458

36. Neelapu SS, Locke FL, Bartlett NL, Lekakis LJ, Miklos DB, Jacobson CA, et al. Axicabtagene Ciloleucel CAR T-Cell Therapy in Refractory Large B-Cell Lymphoma. *The New England journal of medicine*. (2017) 377:2531-44. doi: 10.1056/NEJMoa1707447

37. Neelapu SS, Dickinson M, Munoz J, Ulrickson ML, Thieblemont C, Oluwole OO, et al. Axicabtagene ciloleucel as first-line therapy in high-risk large B-cell lymphoma: the phase 2 ZUMA-12 trial. *Nature medicine*. (2022) 28:735-42. doi: 10.1038/s41591-022-01731-4

38. Pan J, Niu Q, Deng B, Liu S, Wu T, Gao Z, et al. CD22 CAR T-cell therapy in refractory or relapsed B acute lymphoblastic leukemia. *Leukemia*. (2019) 33:2854-66. doi:

10.1038/s41375-019-0488-7

39. Pan J, Tan Y, Wang G, Deng B, Ling Z, Song W, et al. Donor-Derived CD7 Chimeric Antigen Receptor T Cells for T-Cell Acute Lymphoblastic Leukemia: First-in-Human, Phase I Trial. *Journal of clinical oncology : official journal of the American Society of Clinical Oncology*. (2021) 39:3340-51. doi: 10.1200/jco.21.00389

40. Park JH, Romero FA, Taur Y, Sadelain M, Brentjens RJ, Hohl TM, et al. Cytokine Release Syndrome Grade as a Predictive Marker for Infections in Patients With Relapsed or Refractory B-Cell Acute Lymphoblastic Leukemia Treated With Chimeric Antigen Receptor T Cells. *Clin Infect Dis*. (2018) 67:533-40. doi: 10.1093/cid/ciy152

41. Raje N, Berdeja J, Lin Y, Siegel D, Jagannath S, Madduri D, et al. Anti-BCMA CAR T-Cell Therapy bb2121 in Relapsed or Refractory Multiple Myeloma. *The New England journal of medicine*. (2019) 380:1726-37. doi: 10.1056/NEJMoa1817226

42. Ramos CA, Rouce R, Robertson CS, Reyna A, Narala N, Vyas G, et al. In Vivo Fate and Activity of Second- versus Third-Generation CD19-Specific CAR-T Cells in B Cell Non-Hodgkin's Lymphomas. *Molecular therapy : the journal of the American Society of Gene Therapy*. (2018) 26:2727-37. doi: 10.1016/j.ymthe.2018.09.009

43. Ramos CA, Grover NS, Beaven AW, Lulla PD, Wu MF, Ivanova A, et al. Anti-CD30 CAR-T Cell Therapy in Relapsed and Refractory Hodgkin Lymphoma. *Journal of clinical oncology : official journal of the American Society of Clinical Oncology*. (2020) 38:3794-804. doi: 10.1200/jco.20.01342

44. Roddie C, Dias J, O'Reilly MA, Abbasian M, Cadinanos-Garai A, Vispute K, et al. Durable Responses and Low Toxicity After Fast Off-Rate CD19 Chimeric Antigen Receptor-T Therapy in Adults With Relapsed or Refractory B-Cell Acute Lymphoblastic Leukemia. *Journal of clinical oncology : official journal of the American Society of Clinical Oncology*. (2021) 39:3352-63. doi: 10.1200/jco.21.00917

45. Sang W, Shi M, Yang J, Cao J, Xu L, Yan D, et al. Phase II trial of co-administration of CD19- and CD20-targeted chimeric antigen receptor T cells for relapsed and refractory

diffuse large B cell lymphoma. *Cancer medicine*. (2020) 9:5827-38. doi: 10.1002/cam4.3259

46. Sauter CS, Senechal B, Rivière I, Ni A, Bernal Y, Wang X, et al. CD19 CAR T cells following autologous transplantation in poor-risk relapsed and refractory B-cell non-Hodgkin lymphoma. *Blood*. (2019) 134:626-35. doi: 10.1182/blood.2018883421

47. Schuster SJ, Bishop MR, Tam CS, Waller EK, Borchmann P, McGuirk JP, et al. Tisagenlecleucel in Adult Relapsed or Refractory Diffuse Large B-Cell Lymphoma. *The New England journal of medicine*. (2019) 380:45-56. doi: 10.1056/NEJMoa1804980

48. Shah BD, Bishop MR, Oluwole OO, Logan AC, Baer MR, Donnellan WB, et al. KTE-X19 anti-CD19 CAR T-cell therapy in adult relapsed/refractory acute lymphoblastic leukemia: ZUMA-3 phase 1 results. *Blood*. (2021) 138:11-22. doi: 10.1182/blood.2020009098

49. Shah BD, Ghobadi A, Oluwole OO, Logan AC, Boissel N, Cassaday RD, et al. KTE-X19 for relapsed or refractory adult B-cell acute lymphoblastic leukaemia: phase 2 results of the single-arm, open-label, multicentre ZUMA-3 study. *Lancet*. (2021). doi: 10.1016/s0140-6736(21)01222-8. doi: 10.1016/s0140-6736(21)01222-8

50. Shah NN, Highfill SL, Shalabi H, Yates B, Jin J, Wolters PL, et al. CD4/CD8 T-Cell Selection Affects Chimeric Antigen Receptor (CAR) T-Cell Potency and Toxicity: Updated Results From a Phase I Anti-CD22 CAR T-Cell Trial. *Journal of clinical oncology : official journal of the American Society of Clinical Oncology*. (2020) 38:1938-50. doi: 10.1200/jco.19.03279

51. Shah NN, Johnson BD, Schneider D, Zhu F, Szabo A, Keever-Taylor CA, et al. Bispecific anti-CD20, anti-CD19 CAR T cells for relapsed B cell malignancies: a phase 1 dose escalation and expansion trial. *Nature medicine*. (2020) 26:1569-75. doi: 10.1038/s41591-020-1081-3

52. Shalabi H, Martin S, Yates B, Wolters PL, Kaplan C, Smith H, et al. Neurotoxicity Following CD19/CD28ζ CAR T-Cells in Children and Young Adults with B-Cell Malignancies. *Neuro Oncol*. (2022). doi: 10.1093/neuonc/noac034. doi: 10.1093/neuonc/noac034

53. Shi X, Yan L, Shang J, Kang L, Yan Z, Jin S, et al. Anti-CD19 and anti-BCMA CAR T cell

therapy followed by lenalidomide maintenance after autologous stem-cell transplantation for high-risk newly diagnosed multiple myeloma. *Am J Hematol*. (2022) 97:537-47. doi: 10.1002/ajh.26486

54. Siddiqi T, Soumerai JD, Dorritie KA, Stephens DM, Riedell PA, Arnason J, et al. Phase 1 TRANSCEND CLL 004 study of lisocabtagene maraleucel in patients with relapsed/refractory CLL or SLL. *Blood*. (2022) 139:1794-806. doi: 10.1182/blood.2021011895

55. Singh H, Srour SA, Milton DR, McCarty J, Dai C, Gaballa MR, et al. Sleeping beauty generated CD19 CAR T-Cell therapy for advanced B-Cell hematological malignancies. *Frontiers in immunology*. (2022) 13:1032397. doi: 10.3389/fimmu.2022.1032397

56. Spiegel JY, Patel S, Muffly L, Hossain NM, Oak J, Baird JH, et al. CAR T cells with dual targeting of CD19 and CD22 in adult patients with recurrent or refractory B cell malignancies: a phase 1 trial. *Nature medicine*. (2021) 27:1419-31. doi: 10.1038/s41591-021-01436-0

57. Talleur A, Qudiemat A, Métais JY, Langfitt D, Mamcarz E, Crawford JC, et al. Preferential expansion of CD8+ CD19-CAR T cells postinfusion and the role of disease burden on outcome in pediatric B-ALL. *Blood Adv*. (2022). doi: 10.1182/bloodadvances.2021006293. doi: 10.1182/bloodadvances.2021006293

58. Tong C, Zhang Y, Liu Y, Ji X, Zhang W, Guo Y, et al. Optimized tandem CD19/CD20 CAR-engineered T cells in refractory/relapsed B-cell lymphoma. *Blood*. (2020) 136:1632-44. doi: 10.1182/blood.2020005278

59. Turtle CJ, Hanafi LA, Berger C, Gooley TA, Cherian S, Hudecek M, et al. CD19 CAR-T cells of defined CD4+:CD8+ composition in adult B cell ALL patients. *J Clin Invest*. (2016) 126:2123-38. doi: 10.1172/jci85309

60. Wang D, Wang J, Hu G, Wang W, Xiao Y, Cai H, et al. A phase 1 study of a novel fully human BCMA-targeting CAR (CT103A) in patients with relapsed/refractory multiple myeloma. *Blood*. (2021) 137:2890-901. doi: 10.1182/blood.2020008936

61. Wang J, Zhang X, Zhou Z, Liu Y, Yu L, Jia L, et al. A novel adoptive synthetic TCR and antigen receptor (STAR) T-Cell therapy for B-Cell acute lymphoblastic leukemia. *Am J Hematol*. (2022) 97:992-1004. doi: 10.1002/ajh.26586

62. Wang M, Munoz J, Goy A, Locke FL, Jacobson CA, Hill BT, et al. KTE-X19 CAR T-Cell Therapy in Relapsed or Refractory Mantle-Cell Lymphoma. *The New England journal of medicine*. (2020) 382:1331-42. doi: 10.1056/NEJMoa1914347

63. Wang Y, Cao J, Gu W, Shi M, Lan J, Yan Z, et al. Long-Term Follow-Up of Combination of B-Cell Maturation Antigen and CD19 Chimeric Antigen Receptor T Cells in Multiple Myeloma. *Journal of clinical oncology : official journal of the American Society of Clinical Oncology*. (2022) 40:2246-56. doi: 10.1200/jco.21.01676

64. Wei G, Zhang Y, Zhao H, Wang Y, Liu Y, Liang B, et al. CD19/CD22 Dual-Targeted CAR T-cell Therapy for Relapsed/Refractory Aggressive B-cell Lymphoma: A Safety and Efficacy Study. *Cancer Immunol Res*. (2021) 9:1061-70. doi: 10.1158/2326-6066.Cir-20-0675

65. Wu J, Meng F, Cao Y, Zhang Y, Zhu X, Wang N, et al. Sequential CD19/22 CAR T-cell immunotherapy following autologous stem cell transplantation for central nervous system lymphoma. *Blood Cancer J*. (2021) 11:131. doi: 10.1038/s41408-021-00523-2

66. Yan ZX, Li L, Wang W, OuYang BS, Cheng S, Wang L, et al. Clinical Efficacy and Tumor Microenvironment Influence in a Dose-Escalation Study of Anti-CD19 Chimeric Antigen Receptor T Cells in Refractory B-Cell Non-Hodgkin's Lymphoma. *Clin Cancer Res*. (2019) 25:6995-7003. doi: 10.1158/1078-0432.Ccr-19-0101

67. Ying Z, Huang XF, Xiang X, Liu Y, Kang X, Song Y, et al. A safe and potent anti-CD19 CAR T cell therapy. *Nature medicine*. (2019) 25:947-53. doi: 10.1038/s41591-019-0421-7

68. Ying Z, Yang H, Guo Y, Li W, Zou D, Zhou D, et al. Relmacabtagene autoleucel (relma-cel) CD19 CAR-T therapy for adults with heavily pretreated relapsed/refractory large B-cell lymphoma in China. *Cancer medicine*. (2021) 10:999-1011. doi: 10.1002/cam4.3686

69. Ying Z, He T, Jin S, Wang X, Zheng W, Lin N, et al. A durable 4-1BB-based CD19 CAR-T cell for treatment of relapsed or refractory non-Hodgkin lymphoma. *Chinese journal of*

cancer research = Chung-kuo yen cheng yen chiu. (2022) 34:53-62. doi: 10.21147/j.issn.1000-9604.2022.01.05

70. Zhang H, Liu M, Xiao X, Lv H, Jiang Y, Li X, et al. A combination of humanized anti-BCMA and murine anti-CD38 CAR-T cell therapy in patients with relapsed or refractory multiple myeloma. *Leukemia & lymphoma*. (2022) 63:1418-27. doi: 10.1080/10428194.2022.2030476

71. Zhang X, Lu XA, Yang J, Zhang G, Li J, Song L, et al. Efficacy and safety of anti-CD19 CAR T-cell therapy in 110 patients with B-cell acute lymphoblastic leukemia with high-risk features. *Blood Adv*. (2020) 4:2325-38. doi: 10.1182/bloodadvances.2020001466

72. Zhang Y, Li J, Lou X, Chen X, Yu Z, Kang L, et al. A Prospective Investigation of Bispecific CD19/22 CAR T Cell Therapy in Patients With Relapsed or Refractory B Cell Non-Hodgkin Lymphoma. *Front Oncol*. (2021) 11:664421. doi: 10.3389/fonc.2021.664421

73. Zhao WH, Liu J, Wang BY, Chen YX, Cao XM, Yang Y, et al. A phase 1, open-label study of LCAR-B38M, a chimeric antigen receptor T cell therapy directed against B cell maturation antigen, in patients with relapsed or refractory multiple myeloma. *Journal of hematology & oncology*. (2018) 11:141. doi: 10.1186/s13045-018-0681-6

74. Zhou X, Tu S, Wang C, Huang R, Deng L, Song C, et al. Phase I Trial of Fourth-Generation Anti-CD19 Chimeric Antigen Receptor T Cells Against Relapsed or Refractory B Cell Non-Hodgkin Lymphomas. *Frontiers in immunology*. (2020) 11:564099. doi: 10.3389/fimmu.2020.564099

75. Locke FL, Ghobadi A, Jacobson CA, Miklos DB, Lekakis LJ, Oluwole OO, et al. Long-term safety and activity of axicabtagene ciloleucel in refractory large B-cell lymphoma (ZUMA-1): a single-arm, multicentre, phase 1–2 trial. (2019) 20:31-42

76. Bastos-Oreiro M, Gutierrez A, Reguera JL, Iacoboni G, López-Corral L, Terol MJ, et al. Best Treatment Option for Patients With Refractory Aggressive B-Cell Lymphoma in the CAR-T Cell Era: Real-World Evidence From GELTAMO/GETH Spanish Groups. *Frontiers in immunology*. (2022) 13:855730. doi: 10.3389/fimmu.2022.855730

77. Bethge WA, Martus P, Schmitt M, Holtick U, Subklewe M, von Tresckow B, et al. GLA/DRST real-world outcome analysis of CAR T-cell therapies for large B-cell lymphoma in Germany. *Blood*. (2022) 140:349-58. doi: 10.1182/blood.2021015209

78. Gauthier J, Gazeau N, Hirayama AV, Hill JA, Wu V, Cearley A, et al. Impact of CD19 CAR T-cell product type on outcomes in relapsed or refractory aggressive B-NHL. *Blood*. (2022) 139:3722-31. doi: 10.1182/blood.2021014497

79. Ghilardi G, Chong EA, Svoboda J, Wohlfarth P, Nasta SD, Williamson S, et al. Bendamustine is safe and effective for lymphodepletion before tisagenlecleucel in patients with refractory or relapsed large B-cell lymphomas. *Annals of oncology : official journal of the European Society for Medical Oncology*. (2022). doi: 10.1016/j.annonc.2022.05.521. doi: 10.1016/j.annonc.2022.05.521

80. Kuhnl A, Roddie C, Kirkwood AA, Tholouli E, Menne T, Patel A, et al. A national service for delivering CD19 CAR-Tin large B-cell lymphoma - The UK real-world experience. *British journal of haematology*. (2022) 198:492-502. doi: 10.1111/bjh.18209

81. Kwon M, Iacoboni G, Reguera JL, Corral LL, Morales RH, Ortiz-Maldonado V, et al. Axicabtagene ciloleucel compared to tisagenlecleucel for the treatment of aggressive B-cell lymphoma. *Haematologica*. (2022). doi: 10.3324/haematol.2022.280805. doi: 10.3324/haematol.2022.280805

82. Nastoupil LJ, Jain MD, Feng L, Spiegel JY, Ghobadi A, Lin Y, et al. Standard-of-Care Axicabtagene Ciloleucel for Relapsed or Refractory Large B-Cell Lymphoma: Results From the US Lymphoma CAR T Consortium. *Journal of clinical oncology : official journal of the American Society of Clinical Oncology*. (2020) 38:3119-28. doi: 10.1200/jco.19.02104

83. Pasquini MC, Hu ZH, Curran K, Laetsch T, Locke F, Rouce R, et al. Real-world evidence of tisagenlecleucel for pediatric acute lymphoblastic leukemia and non-Hodgkin lymphoma. *Blood Adv*. (2020) 4:5414-24. doi: 10.1182/bloodadvances.2020003092

84. Riedell PA, Hwang WT, Nastoupil LJ, Pennisi M, McGuirk JP, Maziarz RT, et al. Patterns of Use, Outcomes, and Resource Utilization among Recipients of Commercial Axicabtagene

Ciloleucel and Tisagenlecleucel for Relapsed/Refractory Aggressive B Cell Lymphomas. *Transplantation and cellular therapy*. (2022). doi: 10.1016/j.jtct.2022.07.011. doi: 10.1016/j.jtct.2022.07.011

85. Rubin DB, Al Jarrah A, Li K, LaRose S, Monk AD, Ali AB, et al. Clinical Predictors of Neurotoxicity After Chimeric Antigen Receptor T-Cell Therapy. *JAMA neurology*. (2020) 77:1536-42. doi: 10.1001/jamaneurol.2020.2703

86. Strati P, Nastoupil LJ, Westin J, Fayad LE, Ahmed S, Fowler NH, et al. Clinical and radiologic correlates of neurotoxicity after axicabtagene ciloleucel in large B-cell lymphoma. *Blood Adv*. (2020) 4:3943-51. doi: 10.1182/bloodadvances.2020002228

87. Zettler ME, Feinberg BA, Phillips EG, Jr., Klink AJ, Mehta S, Gajra A. Real-world adverse events associated with CAR T-cell therapy among adults age ≥ 65 years. *Journal of geriatric oncology*. (2021) 12:239-42. doi: 10.1016/j.jgo.2020.07.006
